# Maternal Circulating Angiogenic Biomarkers for Predicting Fetal Growth Restriction and Small-for-Gestational-Age Birth Without Preeclampsia

**DOI:** 10.3390/ijms27209155

**Published:** 2026-10-15

**Authors:** Min-A Kim, Eunju Lee, Hye Sun Lee, Ah-Young Choi

**Affiliations:** 1 Department of Obstetrics and Gynecology, Gangnam Severance Hospital, Yonsei University College of Medicine, Seoul 06273, Republic of Korea; cay423@yuhs.ac; 2 Biostatistics Collaboration Unit, Yonsei University College of Medicine, Seoul 06273, Republic of Korea; dmswn1578@yuhs.ac (E.L.); hslee1@yuhs.ac (H.S.L.)

**Keywords:** fetal growth restriction, small for gestational age, placental growth factor, soluble fms-like tyrosine kinase-1, sFlt-1/PlGF ratio

## Abstract

Maternal circulating angiogenic factors are established biomarkers in preeclampsia, but their early profiles in fetal growth impairment without preeclampsia remain unclear. We quantitatively synthesized data from observational studies measuring maternal serum placental growth factor (PlGF), soluble fms-like tyrosine kinase-1 (sFlt-1), or their ratio before 29 weeks and before clinical recognition of growth impairment in singleton pregnancies without preeclampsia, subsequently classified as fetal growth restriction (FGR), small for gestational age (SGA), or unaffected. PubMed, Embase, and the Cochrane Library were searched through April 2026; 22 studies were included. In random-effects meta-analysis, PlGF was lower in affected pregnancies (standardized mean difference [SMD] −0.38; 95% CI −0.63 to −0.13; I^2^ = 92.0%; 19 studies), whereas sFlt-1 did not differ (SMD 0.03; 95% CI −0.27 to 0.34; I^2^ = 79.5%; 12 studies) and the ratio was higher in the primary analysis (SMD 0.39; 95% CI 0.08 to 0.70; I^2^ = 80.0%; seven studies). This pattern suggests that the ratio signal was influenced largely by lower PlGF. Bivariate analysis of five PlGF accuracy studies yielded a summary sensitivity of 0.29 and specificity of 0.86. Despite substantial heterogeneity, reduced PlGF was the most consistent early angiogenic feature in pregnancies subsequently classified as FGR/SGA without preeclampsia. Its low sensitivity does not support its use as a stand-alone screening test, but these findings warrant further evaluation of PlGF within multimarker prediction models.

## 1. Introduction

Fetal growth restriction (FGR) affects approximately 5–10% of pregnancies and remains a leading cause of stillbirth and perinatal morbidity [1,2]. However, many affected fetuses are not identified before birth, and unrecognized FGR is associated with a substantially increased risk of stillbirth [3]. Current detection—largely third-trimester fetal biometry and Doppler assessment—identifies only a proportion of affected pregnancies [2], underscoring the importance of biochemical markers capable of identifying pregnancies at risk earlier in gestation.

FGR describes the failure of a fetus to achieve its biological growth potential [4]. Small-for-gestational-age (SGA) birth, commonly defined as a birthweight below the 10th centile, overlaps with but is not synonymous with pathological FGR [1,4]. A constitutionally small fetus may not have the placental vascular pathology underlying FGR. Nevertheless, many biomarker studies have used SGA as an objectively measurable surrogate for impaired fetal growth, making it necessary to evaluate both outcomes while preserving their conceptual distinction.

Normal placental development requires coordinated trophoblast differentiation, extravillous trophoblast invasion, spiral-artery remodeling, and expansion of the fetoplacental microvascular network. Extravillous trophoblasts acquire an endothelial-like phenotype and replace the endothelium of maternal spiral arteries, transforming high-resistance vessels into high-capacitance channels [5]. In parallel, villous angiogenesis expands the exchange surface required to meet increasing fetal oxygen and nutrient demands [6]. Disruption of either process can impair uteroplacental perfusion and restrict fetal growth, even in the absence of maternal hypertension.

Among circulating indicators of placental function, angiogenic factors have been studied most extensively. Placental growth factor (PlGF), a proangiogenic member of the vascular endothelial growth factor family, is highly expressed in trophoblast and placental endothelial compartments and contributes to placental vascular development [7,8]. Soluble fms-like tyrosine kinase-1 (sFlt-1), generated by alternative splicing of FLT1, is a circulating decoy receptor that binds PlGF and vascular endothelial growth factor and reduces their bioavailability [9,10,11]. Excessive placental production of sFlt-1 produces an antiangiogenic state. Reduced PlGF, increased sFlt-1, and an elevated sFlt-1/PlGF ratio are well-established features of preeclampsia [9,12], and the ratio is used clinically to support short-term risk assessment and management in women with suspected preeclampsia [13,14].

Whether this angiogenic framework extends to impaired fetal growth without preeclampsia remains uncertain. FGR and preeclampsia share features of placental dysfunction, but emerging longitudinal and mechanistic evidence suggests that a pronounced increase in sFlt-1 is more characteristic of the preeclamptic phenotype than of impaired fetal growth alone [15]. In contrast, reduced PlGF may reflect abnormal placental development relevant to both conditions [5,8]. It therefore remains unclear whether impaired fetal growth without preeclampsia is characterized predominantly by reduced PlGF, increased sFlt-1, or alterations in both markers. Accordingly, biomarkers established for preeclampsia may not demonstrate equivalent predictive performance when applied to isolated FGR or SGA.

Previous systematic reviews largely focused on the sFlt-1/PlGF ratio measured in pregnancies with suspected or established FGR. They also included populations in which FGR and preeclampsia were not adequately separated or did not separately synthesize early-pregnancy PlGF, sFlt-1, and the sFlt-1/PlGF ratio [16,17,18]. Interpretation has been further complicated by variation in gestational age at sampling, assay platform, outcome definition, and biomarker threshold [16]. Moreover, the included studies reported either continuous biomarker concentrations or threshold-based diagnostic-accuracy measures, which address related but distinct questions and therefore require separate synthesis frameworks.

We therefore performed a quantitative synthesis of maternal circulating PlGF, sFlt-1, and the sFlt-1/PlGF ratio measured during the first or second trimester, before clinical recognition of fetal growth impairment to characterize early angiogenic alterations preceding FGR or SGA in pregnancies without preeclampsia. We synthesized the evidence using two complementary approaches: standardized differences in biomarker concentrations between affected and unaffected pregnancies and threshold-based diagnostic-accuracy measures where sufficient data were available. Beyond evaluating predictive performance, we examined whether the pooled pattern across PlGF, sFlt-1, and their ratio suggested an early angiogenic profile potentially differing from that typically reported in preeclampsia.

## 2. Results

### 2.1. Included Studies

**Figure 1:**
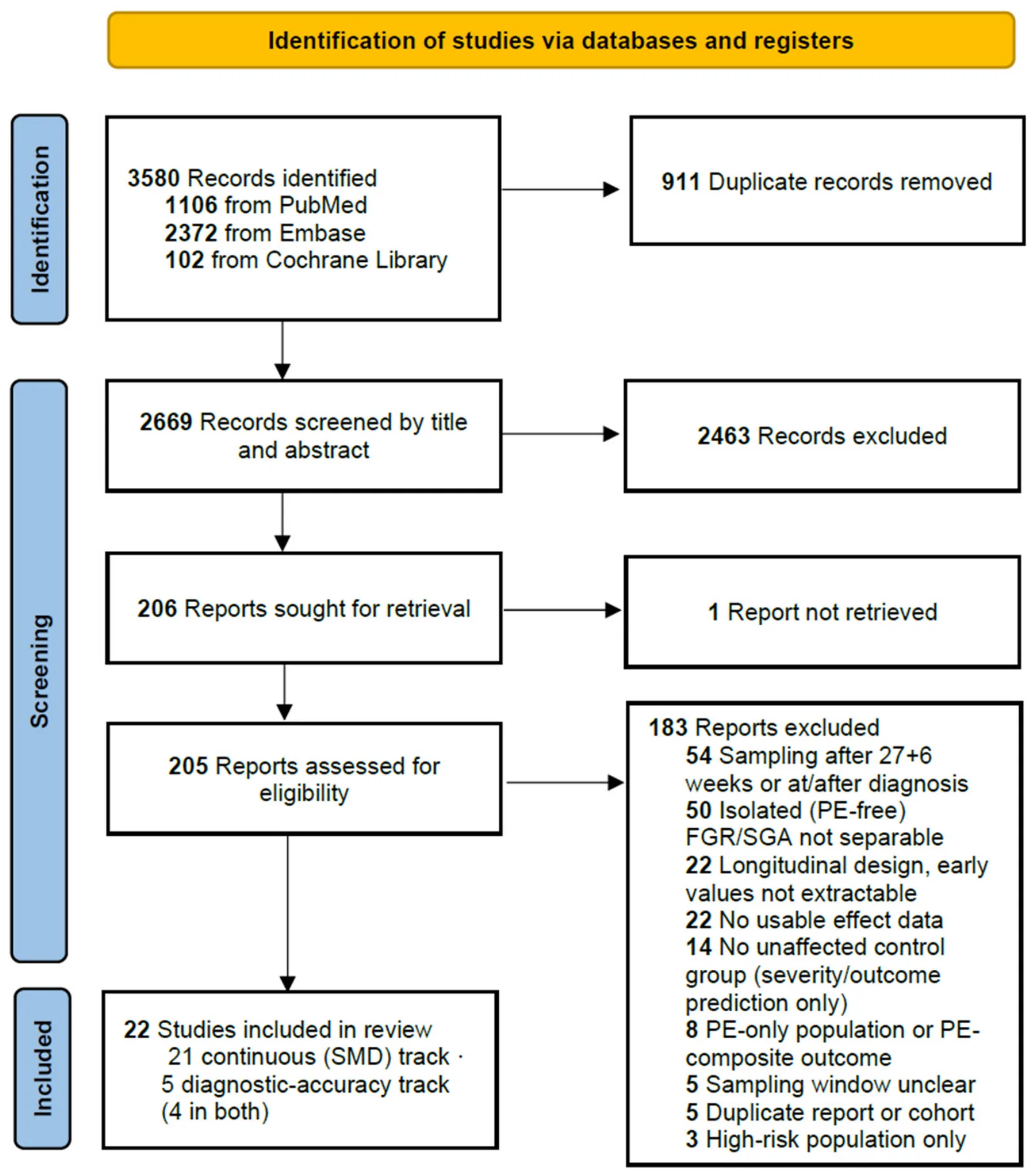
PRISMA 2020 flow diagram of study identification and selection.

The search and selection process is summarized in Figure 1. Of 3580 records identified, 205 full-text articles were assessed for eligibility. Among these, 183 were excluded, most commonly because biomarker measurement occurred outside the predefined gestational window (*n* = 54) or because preeclampsia could not be separated from the growth-restriction outcome group (*n* = 50). The 183 reports excluded after full-text assessment, together with their primary reasons for exclusion, are listed in Supplementary Table S1. Ultimately, 22 studies published between 2001 and 2025 met all eligibility criteria and were included [19,20,21,22,23,24,25,26,27,28,29,30,31,32,33,34,35,36,37,38,39,40]: 21 in the meta-analysis of continuous biomarker levels and 5 in the diagnostic accuracy analysis, with 4 contributing to both (Figure 1). The gestational-age windows at sampling, assay platforms, measurement scales, and summary-statistic formats of the included studies are detailed in Tables S2 and S3; the positivity thresholds used in the diagnostic-accuracy studies are provided in Table S4. Nineteen studies contributed to the PlGF analysis (2702 cases; 19,189 controls), 12 to the sFlt-1 analysis (1800 cases; 10,037 controls), and 7 to the sFlt-1/PlGF ratio analysis (1155 cases; 2393 controls). Study-level effect sizes entering the continuous synthesis are provided in Table S3.

### 2.2. PlGF

**Figure 2:**
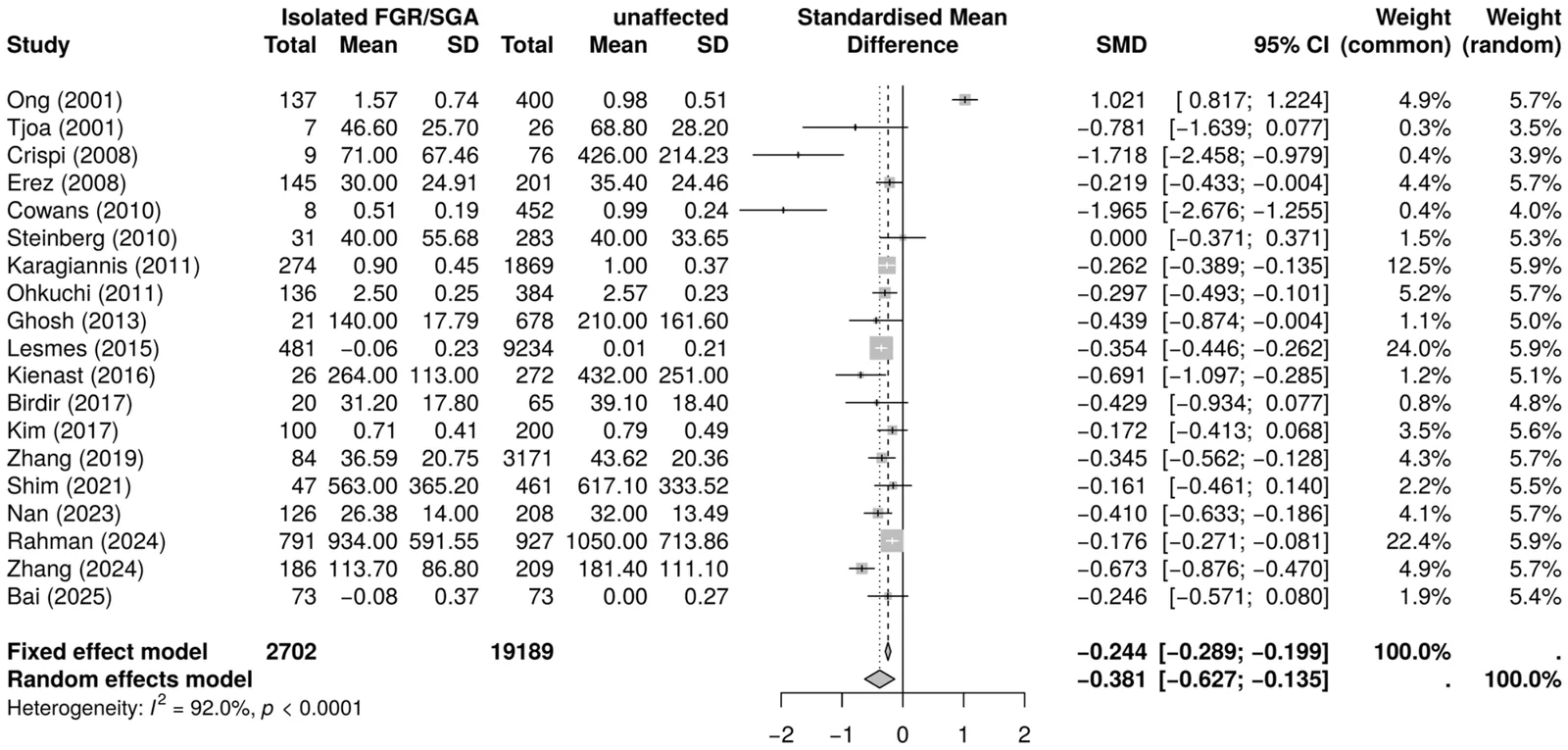
Forest plot of standardized mean differences in maternal PlGF levels measured before 29 weeks of gestation between pregnancies with subsequent isolated FGR/SGA and unaffected pregnancies (k = 19) [19,20,23,24,25,26,27,28,29,30,31,32,34,35,36,37,38,39,40]. The solid vertical line indicates no difference (SMD = 0); the dashed and dotted vertical lines indicate the pooled estimates of the fixed-effect and random-effects models, respectively.

Across 19 studies, PlGF measured before 29 weeks of gestation was lower in pregnancies with subsequent isolated FGR/SGA: random-effects SMD −0.38 (95% CI −0.63 to −0.13; *p* = 0.002), with substantial heterogeneity (I^2^ = 92.0%; Q test *p* < 0.001). Seventeen of the 19 studies had negative point estimates (Figure 2).

### 2.3. sFlt-1

**Figure 3:**
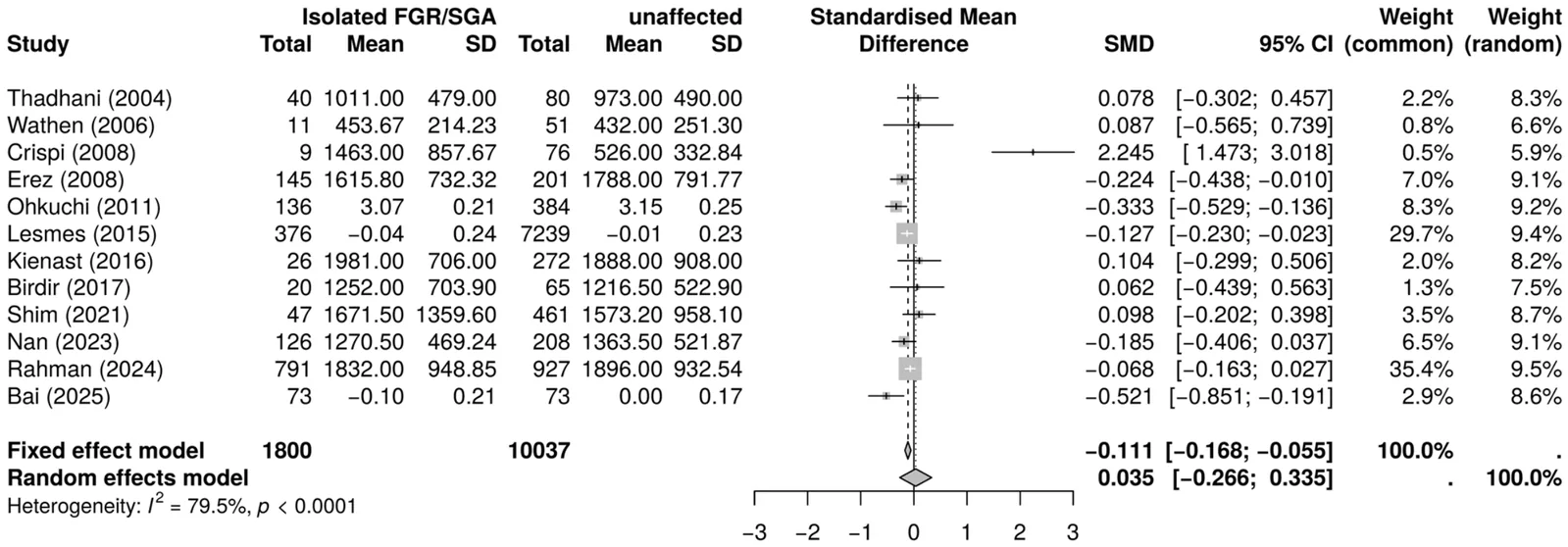
Forest plot of standardized mean differences in maternal sFlt-1 levels measured before 29 weeks of gestation between pregnancies with subsequent isolated FGR/SGA and unaffected pregnancies (k = 12) [21,22,23,24,28,30,31,32,36,37,39,40]. The solid vertical line indicates no difference (SMD = 0); the dashed and dotted vertical lines indicate the pooled estimates of the fixed-effect and random-effects models, respectively.

Across 12 studies, maternal sFlt-1 concentrations showed no statistically significant difference between pregnancies with and without subsequent isolated FGR/SGA under the random-effects model (SMD 0.03, 95% CI −0.27 to 0.34; *p* = 0.82; I^2^ = 79.5%) (Figure 3).

### 2.4. sFlt-1/PlGF Ratio

**Figure 4:**
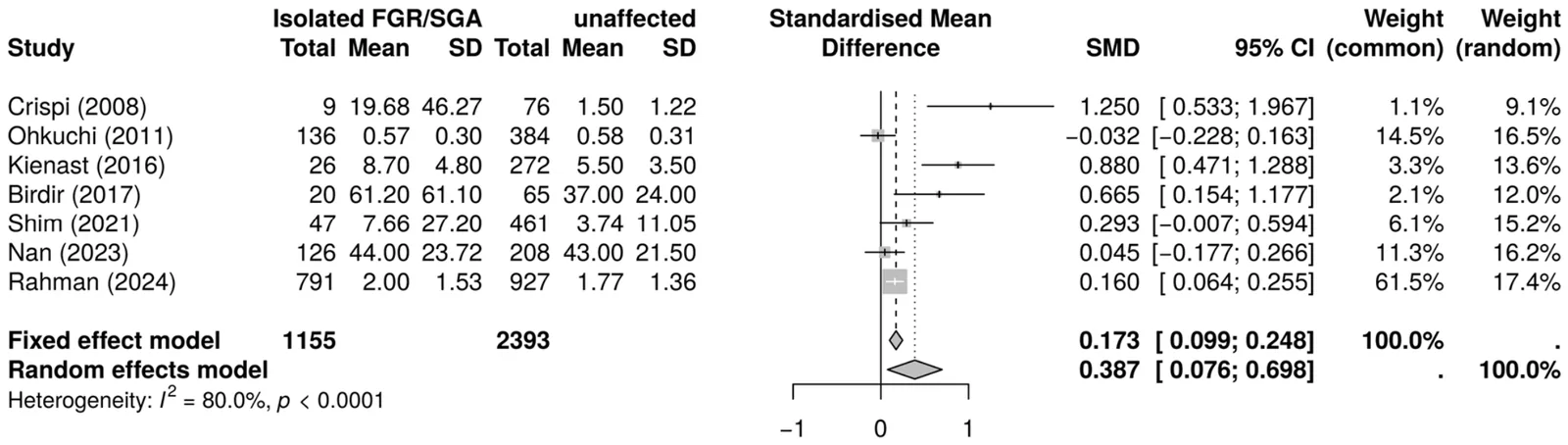
Forest plot of standardized mean differences in the maternal sFlt-1/PlGF ratio measured before 29 weeks of gestation between pregnancies with subsequent isolated FGR/SGA and unaffected pregnancies (k = 7) [23,28,31,32,36,37,39]. The solid vertical line indicates no difference (SMD = 0); the dashed and dotted vertical lines indicate the pooled estimates of the fixed-effect and random-effects models, respectively.

Across seven studies, the sFlt-1/PlGF ratio was significantly higher in pregnancies with subsequent isolated FGR/SGA than in unaffected pregnancies (random-effects SMD 0.39, 95% CI 0.08 to 0.70; *p* = 0.015; I^2^ = 80.0%) (Figure 4). Substantial between-study heterogeneity indicated that the magnitude of this difference varied considerably across studies.

### 2.5. Subgroup and Sensitivity Analyses

For PlGF, no statistically significant between-subgroup differences were observed by assay platform, sampling trimester, outcome family, or smallness criterion (*p* = 0.469, 0.541, 0.791, and 0.182, respectively). Similarly, no significant subgroup differences were observed for sFlt-1 by assay platform, sampling trimester, or outcome family (*p* = 0.498, 0.214, and 0.335, respectively). For the sFlt-1/PlGF ratio, the pooled effect differed by outcome family: the SMD was 0.97 (95% CI 0.62 to 1.33; k = 2) in studies reporting FGR/ IUGR and 0.14 (95% CI 0.01 to 0.27; k = 5) in studies reporting SGA (between-subgroup *p* < 0.001). Because the FGR/IUGR stratum comprised only two studies and the comparison used aggregate study-level data, this finding should be considered exploratory and requires confirmation. No significant subgroup differences were observed by assay platform or sampling trimester (*p* = 0.759 and 0.738, respectively) (Table 1).

In an exploratory analysis stratified by reporting scale, the PlGF estimates were negative and similar in magnitude for raw concentrations (SMD −0.41; k = 12), MoM values (SMD −0.31; k = 4), and log-transformed concentrations (SMD −0.34; k = 3), with no statistically significant between-scale difference (*p* = 0.749). For sFlt-1, the estimates differed between the raw-concentration and log-transformed strata (SMD 0.18 versus −0.29; between-scale *p* = 0.045), although this between-study comparison may reflect other study characteristics in addition to reporting scale. The ratio was not stratified because only one study differed from the predominant reporting scale.

Sensitivity estimates obtained using the DerSimonian–Laird estimator led to the same inferential conclusions as the primary REML estimates for all three markers (Table S5). With Hartung–Knapp-adjusted inference, the PlGF association remained statistically significant (SMD −0.38, 95% CI −0.66 to −0.10; *p* = 0.010), and the sFlt-1 estimate remained non-significant (SMD 0.03, 95% CI −0.35 to 0.42; *p* = 0.848), whereas the confidence interval for the sFlt-1/PlGF ratio included zero (SMD 0.39, 95% CI −0.02 to 0.80; *p* = 0.060).

The average PlGF association retained its negative direction and statistical significance across the leave-one-out and restriction analyses (Tables S6 and S7). In leave-one-out analyses, the pooled estimate ranged from −0.41 to −0.30 across all 19 iterations. The association was also preserved after restriction by sampling window, methodological quality, study design, data-reporting format, and case-group size; exclusion of studies with fewer than 20 affected pregnancies attenuated the pooled SMD to −0.23 (95% CI −0.42 to −0.04; *p* = 0.018; k = 16).

In a sensitivity analysis excluding the two studies whose reported sampling windows extended beyond 27 + 6 weeks, PlGF remained lower in subsequently affected pregnancies (SMD −0.41, 95% CI −0.69 to −0.13; *p* = 0.004; k = 17), sFlt-1 remained nonsignificantly different between groups (SMD 0.06, 95% CI −0.34 to 0.46; *p* = 0.769; k = 10), and the sFlt-1/PlGF ratio remained higher (SMD 0.50, 95% CI 0.03 to 0.97; *p* = 0.038; k = 5), using the primary estimation and inference methods (Table S6).

The sFlt-1/PlGF ratio estimate ranged from 0.29 to 0.47 and remained statistically significant in all leave-one-out iterations under the primary inference method. For sFlt-1, the pooled estimate remained nonsignificant after omission of each study except Crispi et al. [23], whose omission yielded a small negative pooled effect (SMD −0.13, 95% CI −0.23 to −0.04) and reduced heterogeneity from I^2^ = 79.5% to 43.6%; none of the leave-one-out analyses indicated a statistically significant early sFlt-1 elevation.

### 2.6. Meta-Regression

In exploratory random-effects meta-regression, higher study-level mean maternal age was associated with a smaller sFlt-1/PlGF ratio effect (B = −0.09 per year, 95% CI −0.15 to −0.03; *p* = 0.003). Mean maternal age was not significantly associated with the PlGF estimate (B = −0.02, 95% CI −0.13 to 0.08; *p* = 0.636) or the sFlt-1 estimate (B = −0.02, 95% CI −0.06 to 0.02; *p* = 0.411). The association with the ratio effect was based on aggregate data from only seven studies and should therefore be considered hypothesis-generating and potentially unstable. It should not be interpreted as an individual-level effect of maternal age or as evidence that maternal age modifies individual-level biomarker performance.

### 2.7. Diagnostic Accuracy

**Figure 5:**
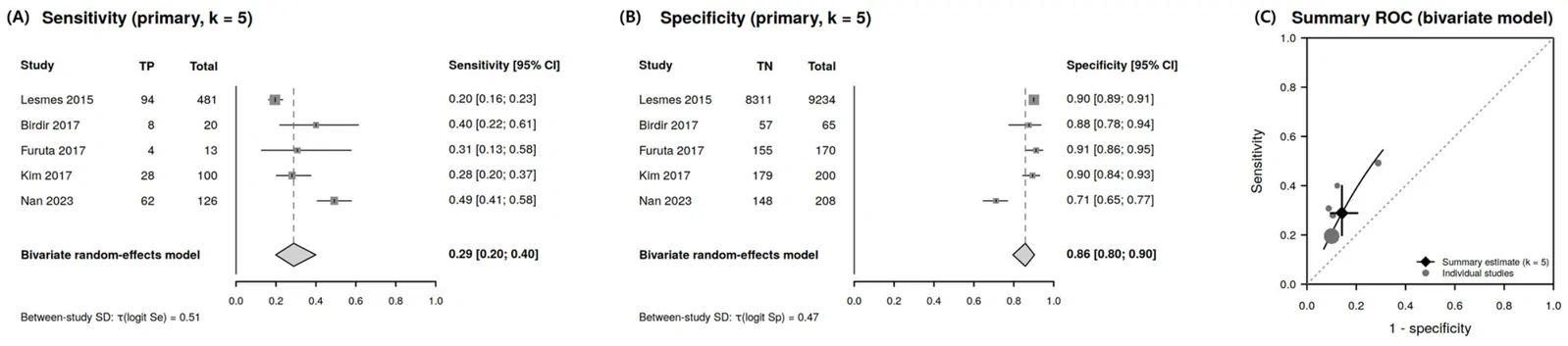
Diagnostic accuracy of maternal PlGF measured before 29 weeks of gestation for isolated FGR/SGA: study-level sensitivity (**A**) and specificity (**B**) with the summary estimate from the bivariate (Reitsma) random-effects model (diamonds), and the summary ROC curve (**C**) [30,32,33,34,37].

Five studies reported PlGF thresholds with complete or reconstructable 2 × 2 data (Table S4). In an exploratory bivariate synthesis, the summary sensitivity was 0.29 (95% CI 0.20 to 0.40) and specificity was 0.86 (95% CI 0.80 to 0.90), with a positive likelihood ratio of 2.03, a negative likelihood ratio of 0.83, and a diagnostic odds ratio of 2.45 (Figure 5). The contributing studies defined positivity using absolute PlGF concentrations, multiples of the median, or gestational-age-specific centiles (Table S4). Accordingly, these estimates summarize performance across study-specific thresholds rather than at a single common PlGF cut-off and should not be regarded as definitive estimates of diagnostic performance. Sensitivity analyses excluding the study judged to be at high risk of bias or the largest contributing study are presented in Table S8. sFlt-1 and the sFlt-1/PlGF ratio were each represented by only two accuracy studies and were therefore not pooled.

### 2.8. Small-Study Effects

Funnel plots showed no marked asymmetry, and Egger regression tests were not statistically significant for PlGF (*p* = 0.408), sFlt-1 (*p* = 0.271), or the sFlt-1/PlGF ratio (*p* = 0.098) (Figure S1). However, funnel-plot asymmetry tests have limited power when few studies are available, particularly for the ratio analysis (k = 7). The nonsignificant results should therefore be considered inconclusive and should not be interpreted as evidence that small-study effects or publication and reporting bias are absent.

### 2.9. Risk of Bias

Newcastle–Ottawa Scale scores among the 22 included studies ranged from 6 to 9 stars. Based on the prespecified threshold of ≥7 stars, 18 studies (82%) were classified as higher-scoring: two studies received 9 stars, three received 8 stars, and 13 received 7 stars; the remaining four received 6 stars (Table S9). Among the five studies contributing to the diagnostic-accuracy analysis, four had no QUADAS-2 domain judged to be at high risk of bias. One study was judged to be at high risk of bias because of concerns in the patient selection domain (Table S10). Because the synthesis included only five studies, this assessment was examined in a sensitivity analysis excluding that study (Table S8).

## 3. Discussion

### 3.1. Principal Findings

In pregnancies destined for growth restriction without preeclampsia, maternal PlGF measured before 29 weeks of gestation was lower than in unaffected pregnancies by approximately 0.4 standard deviations, and 17 of the 19 contributing studies had negative point estimates. In contrast, sFlt-1 showed no early elevation, whereas the sFlt-1/PlGF ratio was modestly higher in the primary analysis, although this finding was sensitive to the method of statistical inference and should be considered exploratory. The higher ratio in the absence of a pooled sFlt-1 elevation is compatible with a contribution from lower PlGF, although the relative contributions of the two components cannot be established from the aggregate data. Together, these findings identify reduced PlGF as the most consistent early circulating angiogenic feature of subsequent isolated FGR/SGA. The absence of a pooled sFlt-1 elevation supports the possibility that its early angiogenic profile differs from that typically observed in preeclampsia.

Most included studies used size-based SGA outcomes, whereas only a minority applied criteria more specific to pathological or placentally mediated FGR. The pooled estimates should therefore be interpreted primarily as associations with subsequent fetal or neonatal smallness and should not be generalized to a homogeneous pathological FGR phenotype.

### 3.2. Comparison with the Existing Literature

Our findings are consistent with longitudinal evidence that the antiangiogenic sFlt-1 surge precedes preeclampsia but is less characteristic of isolated FGR, whereas reduced PlGF reflects deficient placentation shared by both conditions. MacDonald and colleagues reported that increased placental sFLT1 release was a stronger determinant of the maternal circulating angiogenic profile in preeclampsia than in normotensive FGR [15]. Gaccioli and colleagues evaluated ultrasound and the sFlt-1/PlGF ratio in a large prospective nulliparous cohort and provided complementary evidence on the clinical detection of FGR [2]. A recent systematic review of the ratio for distinguishing pathological FGR from constitutional smallness also showed that much of the available evidence was derived from measurements obtained at or after clinical suspicion [16].

Earlier systematic reviews addressed related but clinically distinct questions. Hendrix and colleagues quantitatively pooled PlGF, sFlt-1 and the ratio across gestational-age intervals and found that biomarker differences were generally more pronounced after 26 weeks than in early pregnancy [17]. Interpretation was nevertheless limited by heterogeneous SGA/FGR definitions, sampling windows, assay platforms and cut-offs, and the available data did not permit reliable separation of preeclampsia-associated from normotensive growth disorders. This temporal pattern was directionally consistent with the descriptively larger second-trimester PlGF estimates in our analysis (SMD −0.43 versus −0.28 for first-trimester sampling), although the between-trimester difference was not statistically significant. Similarly, a meta-analysis of the diagnostic capacity of the sFlt-1/PlGF ratio for FGR predominantly pooled measurements obtained at or after clinical suspicion in populations combining FGR with FGR complicated by preeclampsia [18]. The present review extends the literature by restricting sampling before 29 weeks of gestation, before clinical disease, separating growth restriction from preeclampsia, pooling PlGF, sFlt-1 and the ratio separately, and synthesizing continuous and threshold-based evidence in parallel. In this setting, lower PlGF was observed more consistently across studies than higher sFlt-1.

### 3.3. Molecular Mechanisms Underlying the Marker Dissociation

PlGF and sFlt-1 reflect related but distinct aspects of placental pathology. PlGF is a vascular endothelial growth factor-family ligand expressed by trophoblast and placental endothelial compartments. Through VEGFR-1 signaling and interaction with VEGF pathways, it supports endothelial survival, angiogenic branching and placental vascular development [5,6,7]. Placental FGR is associated with defective spiral-artery remodeling, uteroplacental malperfusion, impaired villous angiogenesis and reduced terminal-villus development—stereological hallmarks of growth restriction [5,6]. Consequently, reduced maternal PlGF may reflect limited functional trophoblast mass and deficient development of the placental vascular bed. The early PlGF reduction observed in our pooled analysis is therefore biologically compatible with a primary placental-development phenotype that can precede fetal growth impairment in pregnancies in which preeclampsia is absent.

Circulating sFlt-1 has a different proximate driver. Alternative splicing of FLT1 produces soluble receptor isoforms, including the trophoblast-enriched e15a variant, that bind VEGF and PlGF and reduce their endothelial bioavailability [10,11]. Release of sFlt-1 escalates when the syncytiotrophoblast is stressed by oxidative and endoplasmic-reticulum stress and hypoxia-inducible-factor signaling; in the two-stage model of preeclampsia, this stress response constitutes the second, maternal-facing stage, in which excess sFlt-1 drives the endothelial manifestations of hypertension and proteinuria [10,15,41]. Isolated FGR lacks this overt maternal vascular syndrome, and available longitudinal evidence suggests that any sFlt-1 elevation in normotensive FGR is less marked than that observed in preeclampsia. The absence of an early sFlt-1 elevation in our pooled analysis is therefore consistent with the possibility that impaired placental growth and angiogenesis can occur without the marked maternal-facing antiangiogenic response typical of preeclampsia.

Evidence from other placental disorders further illustrates that the components of the angiogenic axis may dissociate in syndrome-associated patterns. A recent meta-analysis of placental abruption reported higher sFlt-1 and sFlt-1/PlGF ratios without a significant pooled difference in PlGF [42], a pattern distinct from the reduced-PlGF, unchanged-sFlt-1 profile observed here. At the same time, the occurrence of angiogenic abnormalities across different placental complications indicates that these markers are not disease-specific. Circulating profiles may therefore help characterize placental phenotypes, but outcome-specific models are required before they can be used to predict isolated FGR.

The higher sFlt-1/PlGF ratio, together with lower pooled PlGF and no pooled sFlt-1 elevation, is compatible with a contribution from lower PlGF. However, the component and ratio syntheses comprised partly different sets of studies, and the aggregate data do not permit the relative contributions of PlGF and sFlt-1 to the ratio change to be established directly.

The two hypothetical routes to a higher ratio—a fall in PlGF or a rise in sFlt-1—are not necessarily biologically equivalent. A ratio increase occurring alongside lower PlGF without a corresponding sFlt-1 increase may be consistent with impaired placental development, whereas an increase accompanied by higher sFlt-1 may be consistent with a more active antiangiogenic response. Aggregate study-level analyses cannot establish which mechanism operates in individual pregnancies; these interpretations are hypotheses consistent with, rather than demonstrated by, the pooled pattern. Consistent with a phenotype-dependent signal, the ratio effect was markedly larger in the two studies defining the outcome as FGR/IUGR than in those using SGA definitions, and it decreased with higher study-level mean maternal age. Reporting both component concentrations alongside the ratio may help clarify the underlying biomarker patterns, although it cannot by itself establish their mechanisms. Ratio thresholds developed for preeclampsia should not be assumed to have the same performance in early screening for isolated FGR.

### 3.4. Heterogeneity

Substantial heterogeneity was observed for all three markers, likely reflecting variation in study populations, outcome definitions, sampling periods, assay platforms, reporting scales, and data-conversion methods. The subgroup factors reported in Table 1 did not significantly modify the PlGF or sFlt-1 effects, although the absence of significant differences should not be taken as evidence of equivalence, given the sparse strata and limited power of the between-group tests.

The 95% prediction intervals for all three primary analyses included zero (PlGF, −1.50 to 0.74; sFlt-1, −1.12 to 1.19; sFlt-1/PlGF ratio, −0.66 to 1.44; Table S5), indicating that the effect expected in a new comparable setting remains uncertain. For PlGF, heterogeneity decreased when the synthesis was restricted to studies reporting means and standard deviations directly (I^2^ = 57.4%) or when non-nested case–control studies were excluded (I^2^ = 59.1%), suggesting that data-conversion procedures and study-design differences may contribute to the observed variability, although the restricted studies may also differ in other population and design characteristics; heterogeneity in the sFlt-1 and ratio analyses remained largely unexplained.

The larger ratio effect in FGR/IUGR studies than in SGA studies was based on only two FGR/IUGR studies and may also reflect other between-study differences; it should therefore be considered exploratory. The pooled ratio estimate should not be interpreted as a common effect applying equally to FGR/IUGR and SGA.

The inverse study-level association between mean maternal age and the ratio effect may appear counterintuitive because advanced maternal age is a recognized risk factor for placental vascular disorders. However, the meta-regression examined the standardized biomarker difference between groups rather than the risk of developing FGR/SGA. Because this analysis used aggregate data from only seven studies, the finding is hypothesis-generating and should not be interpreted as an individual-level maternal-age effect.

Accordingly, the pooled SMDs should be interpreted as average effects across clinically and methodologically heterogeneous studies rather than as stable effects expected uniformly across clinical settings.

### 3.5. Clinical and Research Implications

The pooled PlGF estimate (SMD −0.38) indicates that PlGF was, on average, approximately 0.4 within-study standard deviations lower in pregnancies subsequently classified as FGR/SGA without preeclampsia than in unaffected pregnancies. However, this average difference alone does not indicate how accurately PlGF can predict FGR/SGA in an individual pregnancy. Because the studies used different assays and reporting scales, the pooled SMD cannot be translated into a universal absolute concentration difference or a clinically actionable PlGF threshold. The continuous synthesis therefore characterizes the early biomarker association, while the separate diagnostic-accuracy synthesis provides complementary evidence on sensitivity and specificity at the thresholds evaluated in the included studies.

Across five diagnostic-accuracy studies comprising 740 affected and 9877 unaffected pregnancies, PlGF showed a summary sensitivity of 29% at 86% specificity. Because the contributing studies used heterogeneous study-specific thresholds, the summary operating point should be considered exploratory; it describes performance across these thresholds and does not identify a common PlGF cut-off that can be recommended for clinical use.

Group-level biomarker differences and threshold-based accuracy estimates do not, by themselves, establish clinical utility. At the summary operating point, most subsequently affected pregnancies would not be identified, while approximately 14% of unaffected pregnancies would be classified as positive. Accordingly, the present findings do not support implementation of PlGF-based screening for isolated FGR/SGA. PlGF should instead be evaluated as a candidate component in the prospective development and external validation of multimarker models for this specific outcome [2,27,35].

Evidence supporting combined first-trimester screening models for preeclampsia or SGA should not be assumed to establish their validity for isolated FGR/SGA without preeclampsia. The present meta-analysis did not evaluate or validate a multimarker prediction algorithm for this specific outcome. Further evaluation of PlGF within such models requires explicit exclusion or separate ascertainment of preeclampsia-associated cases and external validation in the intended population.

Evidence for the diagnostic accuracy of sFlt-1 and the sFlt-1/PlGF ratio was insufficient for pooling, and it remains unclear whether the ratio improves early prediction over PlGF alone. Thresholds developed for preeclampsia should not be extrapolated directly to isolated FGR/SGA. Direct comparison with previous ratio meta-analyses is not appropriate because they evaluated a different biomarker and predominantly included later sampling, clinically suspected or established FGR, or populations in which preeclampsia was not consistently separated [18]. More broadly, the pooled biomarker pattern may be informative as a maternal correlate of placental angiogenic state, even though its stand-alone diagnostic performance is limited.

Future studies should measure PlGF, relevant sFlt-1 isoforms, and both ratio components in the same longitudinal samples, ideally linked to placental histopathology and molecular profiling at delivery. Prospective unselected cohorts should prespecify preeclampsia-free FGR and SGA outcomes, use standardized assays, and report both continuous biomarker distributions and complete 2 × 2 data. Individual-participant-data analyses could enable gestational-age-specific assessment, direct marker comparisons, and evaluation of incremental clinical utility.

### 3.6. Strengths and Limitations

The strengths of this review include restriction to preclinical sampling before 29 weeks of gestation, strict separation of growth restriction from preeclampsia, exclusion of cohorts selected solely on the basis of specific high-risk characteristics—enhancing the applicability of the findings to general obstetric populations—resolution of overlapping cohorts at the source-population level, formal risk-of-bias assessment using both the Newcastle–Ottawa Scale and QUADAS-2, and the use of a bivariate random-effects model for diagnostic-accuracy synthesis. The complementary synthesis of continuous and diagnostic-accuracy data allowed distinction between group-level biomarker differences and threshold-based clinical performance.

Several limitations should be considered. First, heterogeneity was substantial for all three markers. Although exploratory analyses suggested possible contributors—data-conversion procedures and study design for PlGF, and outcome family and maternal age for the ratio—much of the between-study variability remained unexplained. Moreover, the absence of significant subgroup differences should not be interpreted as evidence of equivalence because several strata contained few studies and the analyses had limited power. Second, the evidence base was limited for several analyses: the continuous ratio analysis included only seven studies, its outcome-family comparison was based on two FGR/IUGR studies, diagnostic-accuracy data for sFlt-1 and the ratio were insufficient for pooling, and the PlGF accuracy synthesis was dominated by a single large cohort.

Risk-of-bias assessments also require cautious interpretation. Total NOS scores may conceal limitations within individual domains and should not be interpreted as establishing uniformly high methodological quality; several studies had small case groups, used stored samples, or had design features that may limit representativeness (Tables S2 and S9). The average PlGF association remained negative in analyses restricted to studies with NOS scores ≥ 7 and after exclusion of non-nested case–control studies, although these restrictions do not eliminate all domain-specific risks of bias (Table S6). The diagnostic-accuracy synthesis included only five studies, one of which had an overall high risk of bias and high applicability concerns across multiple QUADAS-2 domains; excluding that study did not materially change the low summary sensitivity (Table S8), and the synthesis remains exploratory.

More generally, the SMD findings reflect average group separation rather than individual predictive performance. Third, some effect sizes were estimated from medians and quartiles. Although SMDs permitted synthesis across different measurement units, they do not eliminate differences in distributional shape associated with MoM standardization or nonlinear transformation. The scale-stratified findings were based on few studies and should not be interpreted as demonstrating equivalence across scales or a causal effect of data transformation. The meta-regression relied on aggregate study-level data and was therefore susceptible to ecological bias. Tests for funnel-plot asymmetry had limited power, particularly for the ratio analysis with only seven studies, and the nonsignificant results do not exclude small-study effects or publication and reporting bias. Finally, because this review did not directly compare isolated FGR/SGA with preeclamptic pregnancies, any proposed distinction between their angiogenic profiles remains an indirect inference based on the pooled pattern and external literature rather than a direct comparative estimate.

## 4. Materials and Methods

### 4.1. Protocol and Registration

This systematic review and meta-analysis was conducted and reported in accordance with the PRISMA 2020 statement [43] and the PRISMA-DTA extension [44]. The protocol was registered with PROSPERO (CRD420261368726).

### 4.2. Eligibility Criteria

We included observational studies of singleton pregnancies in which maternal serum or plasma PlGF, sFlt-1, or the sFlt-1/PlGF ratio was measured during the first trimester (≤13^+6^ weeks) or second trimester (14–27^+6^ weeks), before the clinical diagnosis of fetal growth impairment, and pregnancy outcomes were subsequently classified as FGR, IUGR, SGA, or unaffected. Both cohort and case–control designs were eligible, provided that biomarker sampling preceded the diagnosis or ascertainment of the growth outcome. The target sampling window was ≤27^+6^ weeks. Two studies with reported sampling windows extending marginally beyond this limit, but no later than 28^+6^ weeks, were retained because biomarker measurement preceded ascertainment of the growth outcome (Table S2). The comparator was an unaffected pregnancy group without FGR or SGA drawn from the same source population. Studies restricted to selected high-risk populations—such as cohorts recruited on the basis of pre-existing maternal disorders or abnormal uterine artery Doppler findings—were excluded, so that the included cohorts reflected general obstetric populations. Preeclampsia had to be excluded by study design or the FGR/SGA outcome group without preeclampsia had to be separately extractable. Studies in which preeclampsia could not be excluded or quantitatively separated from the growth-restriction outcome group were excluded.

We excluded multiple pregnancies; studies restricted to third-trimester or diagnostic-time sampling; studies without an unaffected comparator; studies reporting only multivariable-model performance without a marker-specific estimate; and duplicate reports of an already represented cohort. Studies without usable marker-specific data for quantitative synthesis were also excluded. A study could contribute to the continuous synthesis, the diagnostic-accuracy synthesis, or both.

### 4.3. Information Sources and Search Strategy

PubMed/MEDLINE, Embase, and the Cochrane Central Register of Controlled Trials were searched from database inception to 13 April 2026. Controlled vocabulary and free-text terms for PlGF, sFlt-1 or soluble VEGFR-1, angiogenic ratios, and fetal growth outcomes, including FGR, IUGR, SGA, and placental insufficiency, were combined without date, language, or study-design restrictions. The reference lists of included studies and relevant systematic reviews were hand-searched for additional eligible articles. The complete search strategies as executed are provided in Supplementary Appendix S1.

### 4.4. Study Selection and Data Extraction

Two reviewers independently screened titles and abstracts and assessed potentially eligible full-text articles against the prespecified criteria, with disagreements resolved through discussion. Full-text reports unavailable online were requested through institutional document delivery services; one report that could not be obtained was recorded as not retrieved in the PRISMA flow diagram. The two reviewers independently extracted data into a structured workbook, including study design, population and outcome definitions, gestational age at sampling, assay platform, biomarker scale, group sizes, summary statistics, diagnostic thresholds, and 2 × 2 counts. Extracted data were cross-checked against the corresponding tables, figures, and Supplementary Materials.

Publications derived from the same source cohort were not pooled together for the same marker. One representative report was selected for each cohort and marker, and only the earliest eligible sampling time was retained from studies reporting repeated measurements.

### 4.5. Outcome Definitions

The clinical outcome of interest was isolated impaired fetal growth, defined as FGR, IUGR, or SGA occurring in the absence of preeclampsia. Outcome definitions were accepted as specified by each study, most commonly a birthweight or estimated fetal weight below the 10th centile for gestational age, with stricter centile cut-offs or consensus-based FGR criteria applied in a subset of studies; the definition used by each study is listed in Table S2. Because FGR/IUGR and SGA represent overlapping but clinically distinct phenotypes, the combined affected category is clinically heterogeneous. These study-level classifications followed the terminology and operational definitions of the primary studies and did not necessarily distinguish pathological FGR from size-defined SGA.

### 4.6. Statistical Analysis

The primary measure of effect was the standardized mean difference (SMD) [45] in biomarker concentration between pregnancies that subsequently developed isolated FGR, IUGR, or SGA and unaffected pregnancies, calculated as the affected group minus the unaffected group. Accordingly, negative effects for PlGF indicated lower values in affected pregnancies, whereas positive effects for sFlt-1 and the sFlt-1/PlGF ratio indicated higher values in affected pregnancies.

Reported means and standard deviations were used directly, and standard errors were converted to standard deviations. When only medians were reported, the mean was estimated as the median for groups larger than 25, and as (Q1 + median + Q3)/3 for smaller groups when quartiles were available [46,47]; missing standard deviations were estimated as IQR/(2 × 0.6745) [48]. Biomarker values were analyzed on the scale reported by each study. Calculation of an SMD removes the original measurement units but does not harmonize the shapes of the underlying distributions; effects estimated from raw, MoM-standardized, and log-transformed values may therefore represent related but not strictly identical estimands. When a study reported multiple non-overlapping subgroups of the same outcome, subgroup data were combined to generate a single study-level estimate and avoid the inclusion of dependent effect sizes.

Study-specific SMDs were pooled separately for each marker using fixed-effect and random-effects models. For the primary random-effects analyses, between-study variance was estimated using restricted maximum likelihood (REML). Sensitivity analyses used the DerSimonian–Laird estimator [49] and, separately, Hartung–Knapp-adjusted confidence intervals and tests with the REML estimate of between-study variance. Ninety-five percent prediction intervals were calculated for the primary REML syntheses (Table S5). Heterogeneity was assessed using the Cochran Q test and the I^2^ statistic [50]; because substantial heterogeneity was present for all three markers, the random-effects estimates were regarded as primary. Exploratory subgroup analyses examined assay platform, sampling trimester, outcome family (FGR/IUGR versus SGA), and smallness criterion, with between-subgroup difference tests. An exploratory post hoc analysis stratified the syntheses by reporting scale where at least two comparable strata could be formed (Table S6). Exploratory random-effects meta-regression examined associations between study-level mean maternal age and the effect estimates.

Because median- and IQR-based conversions rely on distributional assumptions that may not hold for skewed angiogenic biomarker measurements, a sensitivity analysis was restricted to studies reporting means and standard deviations directly or permitting direct algebraic conversion of a reported standard error to a standard deviation (Table S6). Sensitivity analyses included leave-one-out analyses and restriction of the primary syntheses according to sampling window, methodological quality, study design, data-reporting format, and case-group size. In a post hoc sensitivity analysis, a very small case group was operationally defined as fewer than 20 affected pregnancies. Small-study effects were assessed using funnel plots and the Egger regression test [51].

Studies providing complete or reconstructable 2 × 2 data were synthesized using the Reitsma bivariate random-effects model [52]. Only one operating point per study was included in each analysis: the study’s primary threshold or, failing that, the threshold closest to established clinical cut-offs. Because the included studies used heterogeneous positivity criteria, the summary accuracy estimates describe performance across study-specific thresholds rather than at a single common PlGF cut-off. A continuity correction of 0.5 was applied when required for zero cells. Markers represented by fewer than four studies and reports providing only area under the curve or fixed false-positive-rate results were not quantitatively pooled. Sensitivity analyses of the PlGF diagnostic-accuracy synthesis excluded, in turn, the study judged to be at high risk of bias and the largest contributing study (Table S8).

All tests were two-sided, and *p* values < 0.05 were considered statistically significant. SMD analyses were performed by an academic biostatistics unit using R version 4.6.1 (meta and metafor packages); diagnostic-accuracy analyses were performed in R using the mada package.

### 4.7. Risk-of-Bias Assessment

Risk of bias in studies contributing to the diagnostic-accuracy synthesis was assessed using QUADAS-2 [53], which rates four domains—patient selection, index test, reference standard, and flow and timing—for risk of bias and applicability concerns. Cohort and case–control evidence contributing to the continuous analyses was evaluated using the Newcastle–Ottawa Scale, which assigns up to nine stars across three domains: selection of study groups, comparability of groups, and ascertainment of outcome, applied according to prespecified operational criteria. Studies with a score of 7 stars or higher were classified as higher-scoring for descriptive and sensitivity analyses. Two reviewers independently performed both assessments, with disagreements resolved by discussion.

## 5. Conclusions

Before 29 weeks of gestation and before clinical recognition, reduced maternal PlGF was the most consistent circulating angiogenic feature in pregnancies subsequently classified as FGR/SGA without preeclampsia, whereas sFlt-1 showed no pooled elevation. The sFlt-1/PlGF ratio was modestly increased in the primary analysis, but this finding was sensitive to the method of statistical inference and should be considered exploratory. Because the available evidence predominantly comprised studies using size-based SGA outcomes, these findings should not be generalized to pathological FGR defined by evidence of impaired growth potential or placental dysfunction. The relative contributions of PlGF and sFlt-1 to the ratio finding could not be established from the aggregate data. Although low sensitivity and substantial clinical and methodological heterogeneity do not support PlGF as a stand-alone screening test, these findings warrant further evaluation of PlGF within prospectively developed and externally validated multimarker prediction models.

## Figures and Tables

**Table 1:** Subgroup analyses of the pooled random-effects estimates.

Marker	Subgroup	k	Random-Effects SMD (95% CI)	Between Subgroup *p*
PlGF	Automated clinical platform	10	−0.45 (−0.67 to −0.24)	0.469
	Manual or research assay	9	−0.27 (−0.71 to 0.17)	
	First trimester	9	−0.28 (−0.74 to 0.18)	0.541
	Second trimester	10	−0.43 (−0.62 to −0.24)	
	FGR/IUGR outcome	6	−0.43 (−1.15 to 0.29)	0.791
	SGA outcome	13	−0.33 (−0.45 to −0.22)	
	10th-centile criterion	15	−0.48 (−0.69 to −0.26)	0.182
	5th-centile criterion	3	0.13 (−0.73 to +1.00)	
sFlt-1	Automated clinical platform	6	−0.09 (−0.15 to −0.02)	0.498
	Manual or research assay	6	0.18 (−0.58 to 0.93)	
	First trimester	6	−0.18 (−0.34 to −0.02)	0.214
	Second trimester	6	0.26 (−0.42 to 0.94)	
	FGR/IUGR outcome	4	0.45 (−0.71 to +1.60)	0.335
	SGA outcome	8	−0.12 (−0.20 to −0.05)	
Ratio	Automated clinical platform	5	0.36 (0.06 to 0.65)	0.759
	Manual or research assay	2	0.56 (−0.69 to +1.81)	
	First trimester	2	0.31 (−0.29 to 0.91)	0.738
	Second trimester	5	0.44 (0.02 to 0.85)	
	FGR/IUGR outcome	2	0.97 (0.62 to +1.33)	<0.001
	SGA outcome	5	0.14 (0.01 to 0.27)	

Between-subgroup *p* values were obtained from random-effects tests. Ratio: sFlt-1/PlGF ratio. Smallness-criterion comparisons were limited to PlGF (10th centile, k = 15; 5th centile, k = 3), owing to sparse data for sFlt-1 and the ratio. One PlGF study using another criterion was excluded. This comparison was exploratory because only three studies used the 5th-centile criterion.

## Data Availability

The original contributions presented in this study are included in the article/Supplementary Material. Further inquiries can be directed to the corresponding author.

## Supplementary Materials

The following supporting information can be downloaded at: https://www.mdpi.com/article/10.3390/ijms27209155/s1.
