# Supplementary material for: Maternal Circulating Angiogenic Biomarkers for Predicting Fetal Growth Restriction and Small-for-Gestational-Age Birth Without Preeclampsia
Source: Int J Mol Sci. 2026 Oct 15;27(20):9155. doi: 10.3390/ijms27209155 (PMC13704904; doi:10.3390/ijms27209155)
Supplement: Supplementary file 1 [file ijms-27-09155-s001.zip › ijms-4562663-supplementary.pdf]

Supplementary Materials

Maternal Circulating Angiogenic Biomarkers for Predicting Fetal Growth Restriction and Small-for-Gestational-Age Birth Without Preeclampsia

Contents

Appendix S1. Complete search strategies

Table S1. Reports excluded at full-text assessment, with primary reason for exclusion (n = 183)

Table S2. Characteristics of the 22 included studies

Table S3. Study-level data entering the continuous synthesis (standardized mean differences)

Table S4. Study-level 2 × 2 data for the diagnostic-accuracy synthesis

Table S5. Comparison of random-effects methods and 95% prediction intervals for the primary continuous analyses

Table S6. Restriction sensitivity analyses of the primary continuous syntheses (REML random-effects model)

Table S7. Leave-one-out sensitivity analyses of the primary random-effects (REML) estimates

Table S8. Sensitivity analyses of the bivariate (Reitsma) diagnostic-accuracy synthesis for PlGF

Table S9. Newcastle–Ottawa Scale assessment of the included studies

Table S10. QUADAS-2 assessment of studies contributing to the diagnostic-accuracy synthesis

Figure S1. Funnel plots of the primary continuous analyses

Appendix S1. Complete search strategies

Searches were executed on 13 April 2026.

PubMed/MEDLINE

No.	Query	Results
#6	"Placenta Growth Factor"[Mesh] OR "Vascular Endothelial Growth Factor Receptor-1"[Mesh]	6757
#7	"PlGF"[tiab] OR "placenta growth factor"[tiab] OR "placental growth factor"[tiab] OR "sFlt-1"[tiab] OR "sFlt1"[tiab] OR "soluble fms-like tyrosine kinase"[tiab] OR "soluble fms like tyrosine kinase"[tiab] OR "sVEGFR-1"[tiab] OR "sVEGFR1"[tiab] OR "angiogenic factor*"[tiab] OR "antiangiogenic factor*"[tiab] OR "sFlt-1/PlGF"[tiab] OR "PlGF/sFlt-1"[tiab]	17882
#8	#6 OR #7	20492
#9	"Fetal Growth Retardation"[Mesh]	20369
#10	"IUGR"[tiab] OR "intrauterine growth restriction"[tiab] OR "intrauterine growth retardation"[tiab] OR "fetal growth restriction"[tiab] OR "fetal growth retardation"[tiab] OR "foetal growth restriction"[tiab] OR "FGR"[tiab] OR "small for gestational age"[tiab] OR "SGA"[tiab] OR "placental insufficiency"[tiab] OR "placental dysfunction"[tiab]	46311
#11	#9 OR #10	52634
#12	#8 AND #11	1106

Embase

No.	Query	Results
#1	'placental growth factor'/exp OR 'vasculotropin receptor 1'/exp	19256
#2	'plgf':ti,ab OR 'placenta* growth factor':ti,ab OR 'sflt-1':ti,ab OR 'sflt1':ti,ab OR 'vasculotropin receptor 1':ti,ab OR 'soluble fms-like tyrosine kinase':ti,ab OR 'soluble fms like tyrosine kinase':ti,ab OR 'svegfr-1':ti,ab OR 'svegfr1':ti,ab OR 'angiogenic factor*':ti,ab OR 'antiangiogenic factor*':ti,ab OR 'sflt-1/plgf':ti,ab	26491
#3	#1 OR #2	36980
#4	'intrauterine growth retardation'/exp OR 'placental insufficiency'/exp	72214
#5	'iugr':ti,ab OR 'intrauterine growth restriction':ti,ab OR 'intrauterine growth retardation':ti,ab OR 'fetal growth restriction':ti,ab OR 'fetal growth retardation':ti,ab OR 'foetal growth restriction':ti,ab OR 'fgr':ti,ab OR 'small for gestational age':ti,ab OR 'sga':ti,ab OR 'placental insufficiency':ti,ab OR 'placental dysfunction':ti,ab	71026
#6	#4 OR #5	97850
#7	#3 AND #6	2372

Cochrane Library (CENTRAL)

ID	Search	Results
#1	MeSH descriptor: [Placenta Growth Factor] explode all trees	120
#2	MeSH descriptor: [Vascular Endothelial Growth Factor Receptor-1] explode all trees	120
#3	(PlGF OR "placenta growth factor" OR "placental growth factor"):ti,ab,kw	461
#4	(sFlt-1 OR sFlt1 OR "soluble fms-like tyrosine kinase" OR "soluble fms like tyrosine kinase" OR sVEGFR-1 OR sVEGFR1 OR "soluble flt-1" OR "soluble flt1" OR "VEGFR-1" OR "FLT1"):ti,ab,kw	444
#5	#1 OR #2 OR #3 OR #4	742
#6	MeSH descriptor: [Fetal Growth Retardation] explode all trees	636
#7	(IUGR OR "intrauterine growth restriction" OR "intrauterine growth retardation" OR "fetal growth restriction" OR "fetal growth retardation" OR "foetal growth restriction" OR FGR OR "small for gestational age" OR SGA OR "placental insufficiency" OR "placental dysfunction"):ti,ab,kw	4282
#8	#6 OR #7	4282
#9	#5 AND #8	102

Table S1. Reports excluded at full-text assessment, with primary reason for exclusion (n = 183)

No.	Study (first author, year)	Journal
**Sampling after 27+6 weeks or at/after diagnosis (n = 54)**		
1	Lorenz-Meyer 2026	Journal of Perinatal Medicine
2	Shinohara 2026	Fetal Diagnosis and Therapy
3	Ashwini 2025	Cureus
4	Corker 2025	Journal of Obstetrics and Gynaecology Research
5	Luo 2025	American Journal of Perinatology Reports
6	Dymara-Konopka 2023	International Journal of Molecular Sciences
7	Kluivers 2023	Ultrasound in Obstetrics & Gynecology
8	Bonacina 2022	Journal of Obstetrics and Gynaecology
9	Bonacina 2022	BJOG
10	Di Martino 2022	Nutrients
11	Martín-Palumbo 2022	The Journal of Maternal-Fetal & Neonatal Medicine
12	Mundo 2022	High Altitude Medicine & Biology
13	Garcia-Manau 2021	Acta Obstetricia et Gynecologica Scandinavica
14	Schoots 2021	Placenta
15	Shinar 2021	Pregnancy Hypertension
16	Timokhina 2021	Obstetrics and Gynecology (Russia)
17	Timokhina 2021	Akusherstvo i Ginekologiya (Obstetrics and Gynecology, Moscow)
18	Witwicki 2021	Children (MDPI)
19	Mula 2020	BMC Pregnancy and Childbirth
20	Sharabi-Nov 2020	Fetal Diagnosis and Therapy
21	Sovio 2020	Nature Medicine
22	Anderson 2019	Australian and New Zealand Journal of Obstetrics and Gynaecology
23	Ciobanu 2019	American Journal of Obstetrics and Gynecology
24	Fabjan-Vodusek 2019	Hypertension in Pregnancy
25	Visan 2019	Medicine (Baltimore)
26	Zhang 2019	Journal of Obstetrics and Gynaecology Research
27	Birdir 2018	Pregnancy Hypertension
28	Bligh 2018	Fetal Diagnosis and Therapy
29	He 2018	Frontiers in Endocrinology
30	MacDonald 2018	BMC Pregnancy and Childbirth
31	Muravská 2018	Klinická biochemie a metabolismus (Klin Biochem Metab)
32	Droge 2017	Pregnancy Hypertension
33	Kwiatkowski 2017	Journal of Perinatal Medicine
34	Miranda 2017	Ultrasound in Obstetrics & Gynecology
35	Nanjo 2017	Journal of Obstetrics and Gynaecology Research
36	Margossian 2016	Gynécologie Obstétrique & Fertilité
37	Triunfo 2016	Fetal Diagnosis and Therapy
38	Valino 2016	Ultrasound in Obstetrics & Gynecology
39	Valino 2016	Ultrasound in Obstetrics & Gynecology
40	Zamarian 2016	Archives of Gynecology and Obstetrics
41	Bakalis 2015	Ultrasound in Obstetrics & Gynecology
42	Fadigas 2015	Ultrasound in Obstetrics & Gynecology
43	Gomez-Roig 2015	Gynecologic and Obstetric Investigation
44	Wender-Ozegowska 2015	Hypertension in Pregnancy
45	Alahakoon 2014	The Journal of Maternal-Fetal & Neonatal Medicine
46	Herraiz 2014	Obstetrics & Gynecology
47	Li 2014	Asian Pacific Journal of Tropical Medicine
48	Gomez-Arriaga 2013	Ultrasound in Obstetrics & Gynecology
49	Benton 2012	American Journal of Obstetrics and Gynecology
50	Wallner 2007	Clinical Science
51	Boutsikou 2006	Early Human Development
52	Malamitsi-Puchner 2005	Mediators of Inflammation
53	Shibata 2005	Journal of Clinical Endocrinology & Metabolism
54	Laskowska 2015	Research Journal of Pharmaceutical, Biological and Chemical Sciences
**Isolated (preeclampsia-free) FGR/SGA not separable (n = 50)**		
55	Creswell 2026	American Journal of Obstetrics & Gynecology
56	Golob 2026	American Journal of Obstetrics & Gynecology MFM
57	Torres-Torres 2026	International Journal of Gynecology & Obstetrics
58	Youssef 2026	Ultrasound Obstet Gynecol
59	Das 2025	International Journal of Clinical Obstetrics and Gynaecology
60	Huang 2025	International Journal of Women's Health
61	Li 2025	Frontiers in Endocrinology
62	Martin-Alonso 2025	Medicina (MDPI)
63	Mojumder 2025	International Journal of Clinical Obstetrics and Gynaecology
64	Tang 2025	Frontiers in Pediatrics
65	Zhou 2025	Frontiers in Immunology
66	Balyan 2024	International Journal of Gynecology & Obstetrics
67	Han 2024	Clinical Medicine of China
68	Magee 2024	BJOG (British Journal of Obstetrics and Gynaecology)

69	Nguyen-Hoang 2024	Ultrasound in Obstetrics & Gynecology
70	Papastefanou 2024	Ultrasound in Obstetrics & Gynecology
71	Sovio 2024	BJOG (British Journal of Obstetrics and Gynaecology)
72	Zheng 2024	Ultrasound in Obstetrics & Gynecology
73	Alvarez 2023	Pregnancy Hypertension
74	Ashwal 2023	Prenatal Diagnosis
75	Mula 2023	International Journal of Gynecology & Obstetrics
76	Serrano 2023	International Journal of Gynecology & Obstetrics
77	Tousty 2023	Journal of Clinical Medicine
78	Kudryavtseva 2022	Akusherstvo, Ginekologia i Reprodukcia
79	Mabulabwalya 2022	International Journal of Gynecology & Obstetrics
80	Parry 2022	American Journal of Obstetrics and Gynecology
81	Audette 2021	Journal of Obstetrics and Gynaecology Canada
82	Boutin 2021	American Journal of Obstetrics & Gynecology
83	Hanchard 2021	Australasian Journal of Ultrasound in Medicine
84	Hu 2021	Ultrasound in Obstetrics & Gynecology
85	Nowacka 2021	Journal of Clinical Medicine
86	Noël 2021	Ultrasound in Obstetrics & Gynecology
87	Papastefanou 2021	Ultrasound in Obstetrics & Gynecology
88	Romero-Infante 2021	Journal of Obstetrics and Gynaecology Research
89	Hendrix 2019	Fetal Diagnosis and Therapy
90	Allen 2018	Journal of Maternal-Fetal & Neonatal Medicine
91	Litwinska 2017	Advances in Clinical and Experimental Medicine
92	Sung 2017	Obstetrics & Gynecology Science
93	Triunfo 2017	Ultrasound in Obstetrics & Gynecology
94	Crovetto 2016	Ultrasound in Obstetrics & Gynecology
95	Litwinska 2015	Ginekologia Polska
96	Nucci 2014	Fetal Diagnosis and Therapy
97	Schwartz 2014	American Journal of Obstetrics and Gynecology
98	Boucoiran 2013	American Journal of Perinatology
99	Elkholi 2013	Middle East Fertility Society Journal
100	Coolman 2012	Obstetrics & Gynecology
101	Vandenberghe 2011	Prenatal Diagnosis
102	Poon 2008	Prenatal Diagnosis
103	Espinoza 2007	American Journal of Obstetrics and Gynecology
104	Smith 2007	Obstetrics & Gynecology
**Longitudinal design, early values not extractable (n = 22)**		
105	Di Martino 2025	Best Practice & Research Clinical Obstetrics & Gynaecology
106	Figueiro 2025	Journal of Obstetrics and Gynaecology Canada (JOGC)
107	Jungelson 2025	Hypertension
108	Kumar 2024	Placenta
109	Dathan-Stumpf 2022	American Journal of Obstetrics & Gynecology
110	Iannaccone 2022	Hypertension in Pregnancy
111	Kumar 2022	Pregnancy Hypertension
112	Lorenz-Meyer 2022	Pregnancy Hypertension
113	Arenas 2021	American Journal of Obstetrics & Gynecology
114	Bækgaard-Thorsen 2020	Journal of Maternal-Fetal & Neonatal Medicine
115	Benton 2016	Placenta
116	Griffin 2015	Ultrasound in Obstetrics & Gynecology
117	Borras 2014	Journal of Obstetrics and Gynaecology
118	Schoofs 2014	Journal of Perinatal Medicine
119	Chaiworapongsa 2008	The Journal of Maternal-Fetal & Neonatal Medicine
120	Romero 2008	The Journal of Maternal-Fetal & Neonatal Medicine
121	Semczuk-Sikora 2007	Ginekologia Polska
122	Stepan 2007	Journal of Clinical Endocrinology & Metabolism
123	Crispi 2006	American Journal of Obstetrics and Gynecology
124	Bersinger 2004	Acta Obstetricia et Gynecologica Scandinavica
125	Taylor 2003	American Journal of Obstetrics and Gynecology
126	Helske 2001	Molecular Human Reproduction
**No usable effect data (n = 22)**		
127	Chaveeva 2025	Ultrasound in Obstetrics & Gynecology
128	Maggi 2025	Pregnancy Hypertension: An International Journal of Women's Cardiovascular Health
129	Papastefanou 2024	Ultrasound in Obstetrics & Gynecology
130	Gaccioli 2023	Hypertension
131	Kapustin 2023	Journal of Obstetrics and Women's Diseases
132	Palma dos Reis 2023	Acta Obstetricia et Gynecologica Scandinavica
133	Mendoza 2022	International Journal of Gynecology & Obstetrics
134	Verma 2022	The Journal of Obstetrics and Gynecology of India
135	McLaughlin 2021	Hypertension
136	Mendoza 2021	Acta Obstetricia et Gynecologica Scandinavica
137	Antunes 2020	Journal of Perinatal Medicine
138	Simon 2020	Pregnancy Hypertension
139	Herraiz 2018	Pregnancy Hypertension

140	Honigberg 2018	Journal of Perinatology
141	Komwilaisak 2017	Journal of the Medical Association of Thailand
142	McCowan 2017	PLOS ONE
143	Poon 2015	Ultrasound in Obstetrics & Gynecology
144	Mijal 2012	The Journal of Maternal-Fetal & Neonatal Medicine
145	Åsvold 2011	American Journal of Epidemiology
146	Savvidou 2008	Ultrasound in Obstetrics & Gynecology
147	Stepan 2007	Hypertension
148	Savvidou 2006	American Journal of Obstetrics and Gynecology
**No unaffected control group (severity/outcome prediction only) (n = 14)**		
149	Armengol-Alsina 2026	Ultrasound in Obstetrics & Gynecology
150	Bartels 2026	American Journal of Obstetrics and Gynecology
151	Bonacina 2026	Ultrasound in Obstetrics & Gynecology
152	Arnouts 2025	BMC Pregnancy Childbirth
153	Hughes 2025	Australian and New Zealand Journal of Obstetrics and Gynaecology
154	Kamphof 2025	Ultrasound in Obstetrics & Gynecology
155	Kosinska-Kaczynska 2025	The Journal of Maternal-Fetal & Neonatal Medicine
156	Prins 2025	European Journal of Obstetrics & Gynecology and Reproductive Biology
157	Youssef 2025	Ultrasound in Obstetrics & Gynecology
158	Zhao 2025	BMC Pregnancy Childbirth
159	Hong 2024	BJOG (British Journal of Obstetrics and Gynaecology)
160	Hong 2024	Ultrasound in Obstetrics & Gynecology
161	Rajiv 2024	AJOG Global Reports
162	Swissa 2024	American Journal of Perinatology
**Preeclampsia-only population or preeclampsia-composite outcome (n = 8)**		
163	Cote 2025	Journal of Obstetrics and Gynaecology Canada
164	Linares 2025	European Journal of Obstetrics & Gynecology and Reproductive Biology
165	Ling 2025	The Journal of Maternal-Fetal & Neonatal Medicine
166	Lykou 2025	Hypertension in Pregnancy
167	Anto 2024	Placenta
168	Malladad 2024	International Journal of Academic Medicine and Pharmacy
169	Katz 2022	Ha-Refuah
170	Graupner 2020	Archives of Gynecology and Obstetrics
**Sampling window unclear (n = 5)**		
171	Giorgione 2024	European Journal of Obstetrics & Gynecology and Reproductive Biology
172	Gladstone 2024	JAMA Network Open
173	Nikita 2024	International Journal of Academic Medicine and Pharmacy
174	Kwiatkowski 2021	Pregnancy Hypertension
175	Gaccioli 2018	Lancet Child & Adolescent Health
**Duplicate report or cohort (n = 5)**		
176	Cote 2024	Journal of Obstetrics and Gynaecology Canada
177	Dymara-Konopka 2023	Journal of Clinical Medicine
178	Papastefanou 2022	Ultrasound in Obstetrics & Gynecology
179	Margossian 2015	Gynecologie Obstetrique Fertilite
180	Poon 2013	Fetal Diagnosis and Therapy
**High-risk population only (n = 3)**		
181	Gupta 2026	Journal of Obstetrics and Gynecology of India
182	Raia-Barjat 2019	Journal of Maternal-Fetal & Neonatal Medicine
183	Diab 2008	International Journal of Gynecology & Obstetrics

Reports are grouped according to the primary reason for exclusion used in the PRISMA flow diagram (Figure 1). When more than one exclusion criterion applied, one primary reason was assigned to maintain mutually exclusive categories; additional applicable reasons are not represented in the category totals.

Table S2. Characteristics of the 22 included studies

Study	Country	Design	Cases / Controls	GA at sampling	Marker(s)	Outcome definition	Assay platform	Track; analysis set	PE-free status
Ong 2001 [19]	UK	Case–control (stored sera)	137 / 400	11–14 wk	PlGF	FGR: BW <5th centile, PE-free	Manual ELISA	Continuous; primary	Separate reporting
Tjoa 2001 [20]	Netherlands	Longitudinal cohort (retrosp. analysis)	7 / 26	14–17 wk	PlGF	IUGR, PE-free (centile n.s.)	Manual ELISA	Continuous; primary	Explicit exclusion
Thadhani 2004 [21]	USA	Nested case–control (MOMS)	40 / 80	~10 wk	sFlt-1	SGA: BW <10th centile	Manual ELISA	Continuous; primary	Separate reporting
Wathen 2006 [22]	Finland	Case–control (stored sera)	11 / 51	13.7 / 19.2 wk	sFlt-1	IUGR without PE (composite)	Manual ELISA	Continuous; primary	Separate reporting
Erez 2008 [24]	Chile / USA	Longitudinal case–control	145 / 201	6–15 wk	PlGF, sFlt-1	SGA: BW <10th centile	Manual ELISA	Continuous; primary	Separate reporting
Crispi 2008 [23]	Spain	Nested case–control	9 / 76	24 wk	PlGF, sFlt-1, ratio	IUGR: BW <10th + UA Doppler	Manual ELISA	Continuous; primary	Separate reporting
Steinberg 2010 [26]	USA	Nested case–control (MOMS)	31 / 283	11–13 wk	PlGF	SGA: BW <10th centile, PE-free	Manual ELISA	Continuous; primary	Explicit exclusion
Cowans 2010 [25]	Germany / UK	Matched case–control	8 / 452	11^{+0}–13^{+6} wk	PlGF	SGA: BW <10th centile, PE-free	Automated platform	Continuous; primary	Separate reporting
Karagiannis 2011 [27]	UK	Prospective screening cohort	274 / 1,869	11–13 wk	PlGF	SGA: BW <5th centile, PE-free	Manual ELISA	Continuous; primary	Explicit exclusion
Ohkuchi 2011 [28]	Japan	Prospective cohort	136 / 384	19–25 wk	PlGF, sFlt-1, ratio	SGA: BW <10th centile, PE-free	Manual ELISA	Continuous; primary	Explicit exclusion
Ghosh 2013 [29]	India	Prospective cohort	21 / 678	20–22 wk	PlGF	Early-onset IUGR: EFW/AC <10th	Manual ELISA	Continuous; primary	Separate reporting
Lesmes 2015 [30]	UK	Prospective screening cohort	481 / 9,234	19–24 wk	PlGF, sFlt-1	SGA: BW <5th centile, PE-free	Automated platform	Cont. + DTA; primary	Explicit exclusion
Kienast 2016 [31]	Ecuador	Prospective cohort	26 / 272	18–25 wk	PlGF, sFlt-1, ratio	FGR: BW <10th centile, PE-free	Automated platform	Continuous; primary	Separate reporting
Birdir 2017 [32]	Germany	Prospective cohort	20 / 65	11–13 wk	PlGF, sFlt-1, ratio	SGA: customised BW <10th, PE/PIH-free	Automated platform	Cont. + DTA; primary	Explicit exclusion
Furuta 2017 [33]	Japan	Prospective cohort	13 / 170	24–27 wk	PlGF, ratio	SGA (delivery ≥36 wk), HTN/GDM excluded	Automated platform	DTA only; primary	Normotensive cohort
Kim 2017 [34]	South Korea	Prospective cohort	100 / 200	15–18 wk	PlGF	SGA: BW <10th centile, PE-free	Automated platform	Cont. + DTA; primary	Explicit exclusion
Zhang 2019 [35]	China	Prospective screening cohort	84 / 3,171	11^{+0}–13^{+6} wk	PlGF	SGA: BW <10th centile, PE-free	Automated platform	Continuous; primary	Separate reporting
Shim 2021 [36]	South Korea	Prospective cohort	47 / 461	20^{+0}–28^{+6} wk	PlGF, sFlt-1, ratio	SGA: BW <10th centile, PE excluded post hoc	Automated platform	Continuous; primary	Explicit exclusion
Nan 2023 [37]	Spain	Matched case–control	126 / 208	9–13 wk	PlGF, sFlt-1, ratio	SGA: BW <10th centile, PE-free	Automated platform	Cont. + DTA; primary	Separate reporting
Zhang 2024 [38]	China	Matched case–control	186 / 209	14–20 wk	PlGF	SGA: BW <10th centile for GA and sex	Automated platform	Continuous; primary	Separate reporting
Rahman 2024 [39]	Bangladesh	Population-based cohort (AMANHI)	791 / 927	24–28 wk	PlGF, sFlt-1, ratio	SGA: BW <10th centile	Automated platform	Continuous; primary	Explicit exclusion
Bai 2025 [40]	Hong Kong, China	Nested case–control	73 / 73	11^{+0}–13^{+6} wk	PlGF, sFlt-1	FGR: Delphi consensus criteria	Manual multiplex/research	Continuous; primary	Explicit exclusion

PE-free status: 'Explicit exclusion' indicates that preeclampsia cases were excluded from the cohort or from the FGR/SGA outcome group by design; 'Normotensive cohort' indicates restriction to pregnancies without hypertensive disorders; 'Separate reporting' indicates that preeclampsia was analyzed as a separate outcome group or that the FGR/SGA group without preeclampsia was reported separately. GA, gestational age; wk, weeks; BW, birthweight; EFW, estimated fetal weight; AC, abdominal circumference; UA, umbilical artery; PE, preeclampsia; PIH, pregnancy-induced hypertension; HTN, hypertension; GDM, gestational diabetes; n.s., not specified; Cont., continuous (SMD) track; DTA, diagnostic test accuracy track.

MOMS cohort represented once per marker (PlGF: Steinberg 2010; sFlt-1: Thadhani 2004). The King's College 11–13-week cohort is represented by Karagiannis 2011 in the continuous track. Erez 2008, Wathen 2006, Steinberg/Tjoa first/second-trimester repeat samples: the earliest eligible time point is used in the primary analysis. Reference numbers in square brackets correspond to the reference list of the main text.

Group sizes in this table describe the listed study groups; marker-specific sample sizes are given in Table S3.

Table S3. Study-level data entering the continuous synthesis (standardized mean differences)

Marker	Study	Cases (n)	Controls (n)	SMD	95% CI	Scale/unit	Summary statistics as reported	Assay platform (analysis group)	Analysis set
PlGF	Ong 2001 [19]	137	400	1.021	0.818 to 1.224	MoM	Median_SD	Manual or research-use assay	Primary
PlGF	Tjoa 2001 [20]	7	26	-0.781	-1.618 to 0.056	ng/L	Mean_SD	Manual or research-use assay	Primary
PlGF	Crispi 2008 [23]	9	76	-1.683	-2.414 to -0.952	pg/mL	Median_IQR_width	Manual or research-use assay	Primary
PlGF	Erez 2008 [24]	145	201	-0.118	-0.331 to 0.095	pg/mL	Median_IQR	Manual or research-use assay	Primary
PlGF	Cowans 2010 [25]	8	452	-1.955	-2.665 to -1.246	MoM	Median_IQR_width	Automated IVD clinical platform	Primary
PlGF	Steinberg 2010 [26]	31	283	0	-0.370 to 0.370	pg/mL	Mean_SEM_to_SD	Manual or research-use assay	Primary
PlGF	Karagiannis 2011 [27]	274	1869	-0.252	-0.379 to -0.125	MoM	Median_IQR	Manual or research-use assay	Primary
PlGF	Ohkuchi 2011 [28]	136	384	-0.297	-0.493 to -0.101	log10 pg/mL	Mean_SD	Manual or research-use assay	Primary
PlGF	Ghosh 2013 [29]	21	678	-0.635	-1.070 to -0.200	pg/mL	Median_IQR	Manual or research-use assay	Primary
PlGF	Lesmes 2015 [30]	481	9234	-0.354	-0.446 to -0.262	log10 MoM	Mean_SD_derived_pool (early- and term-SGA subgroups combined)	Automated IVD clinical platform	Primary
PlGF	Kienast 2016 [31]	26	272	-0.691	-1.096 to -0.286	pg/mL	Mean_SD	Automated IVD clinical platform	Primary
PlGF	Birdir 2017 [32]	20	65	-0.429	-0.930 to 0.072	pg/mL	Mean_SD	Automated IVD clinical platform	Primary
PlGF	Kim 2017 [34]	100	200	-0.388	-0.629 to -0.146	MoM	Median_IQR	Automated IVD clinical platform	Primary
PlGF	Zhang 2019 [35]	84	3171	-0.294	-0.511 to -0.077	pg/mL	Median_IQR	Automated IVD clinical platform	Primary
PlGF	Shim 2021 [36]	47	461	-0.161	-0.460 to 0.139	pg/mL	Mean_SD	Automated IVD clinical platform	Primary
PlGF	Nan 2023 [37]	126	208	-0.285	-0.507 to -0.063	pg/mL	Median_IQR	Automated IVD clinical platform	Primary
PlGF	Rahman 2024 [39]	791	927	-0.21	-0.305 to -0.115	pg/mL	Median_IQR	Automated IVD clinical platform	Primary
PlGF	Zhang 2024 [38]	186	209	-0.673	-0.876 to -0.470	pg/mL	Mean_SD	Automated IVD clinical platform	Primary
PlGF	Bai 2025 [40]	73	73	-0.246	-0.570 to 0.078	log10 MoM	Mean_SD	Manual or research-use assay	Primary
sFlt-1	Thadhani 2004 [21]	40	80	0.078	-0.300 to 0.455	pg/mL	Mean_SD	Manual or research-use assay	Primary
sFlt-1	Wathen 2006 [22]	11	51	0.143	-0.501 to 0.787	pg/mL	Median_IQR	Manual or research-use assay	Primary
sFlt-1	Crispi 2008 [23]	9	76	2.062	1.309 to 2.815	pg/mL	Median_IQR_width	Manual or research-use assay	Primary
sFlt-1	Erez 2008 [24]	145	201	-0.124	-0.337 to 0.090	pg/mL	Median_IQR	Manual or research-use assay	Primary
sFlt-1	Ohkuchi 2011 [28]	136	384	-0.333	-0.529 to -0.136	log10 pg/mL	Mean_SD	Manual or research-use assay	Primary
sFlt-1	Lesmes 2015 [30]	376	7239	-0.127	-0.230 to -0.023	log10 MoM	Mean_SD_derived_pool (early- and term-SGA subgroups combined)	Automated IVD clinical platform	Primary
sFlt-1	Kienast 2016 [31]	26	272	0.104	-0.297 to 0.505	pg/mL	Mean_SD	Automated IVD clinical platform	Primary
sFlt-1	Birdir 2017 [32]	20	65	0.062	-0.435 to 0.559	pg/mL	Mean_SD	Automated IVD clinical platform	Primary
sFlt-1	Shim 2021 [36]	47	461	0.098	-0.202 to 0.398	pg/mL	Mean_SD	Automated IVD clinical platform	Primary
sFlt-1	Nan 2023 [37]	126	208	-0.309	-0.531 to -0.087	pg/mL	Median_IQR	Automated IVD clinical platform	Primary
sFlt-1	Rahman 2024 [39]	791	927	-0.058	-0.153 to 0.037	pg/mL	Median_IQR	Automated IVD clinical platform	Primary
sFlt-1	Bai 2025 [40]	73	73	-0.521	-0.849 to -0.192	log10 MoM	Mean_SD	Manual or research-use assay	Primary
sFlt-1/PlGF ratio	Crispi 2008 [23]	9	76	1.06	0.357 to 1.764	unitless	Median_IQR_width	Manual or research-use assay	Primary
sFlt-1/PlGF ratio	Ohkuchi 2011 [28]	136	384	-0.032	-0.228 to 0.163	log10 ratio	Mean_SD	Manual or research-use assay	Primary
sFlt-1/PlGF ratio	Kienast 2016 [31]	26	272	0.88	0.472 to 1.287	unitless	Mean_SD	Automated IVD clinical platform	Primary
sFlt-1/PlGF ratio	Birdir 2017 [32]	20	65	0.665	0.159 to 1.172	unitless	Mean_SD	Automated IVD clinical platform	Primary
sFlt-1/PlGF ratio	Shim 2021 [36]	47	461	0.293	-0.007 to 0.593	unitless	Mean_SD	Automated IVD clinical platform	Primary
sFlt-1/PlGF ratio	Nan 2023 [37]	126	208	-0.002	-0.223 to 0.219	unitless	Median_IQR	Automated IVD clinical platform	Primary
sFlt-1/PlGF ratio	Rahman 2024 [39]	791	927	0.159	0.064 to 0.254	unitless	Median_IQR	Automated IVD clinical platform	Primary

SMD, standardized mean difference (Hedges g, affected minus unaffected), as displayed in the forest plots (main text). All listed studies contributed to the primary quantitative synthesis.

Table S4. Study-level 2 × 2 data for the diagnostic-accuracy synthesis

Study	Marker	Threshold	TP	FP	FN	TN	Role in synthesis
Nan 2023 [37]	PlGF	<25.87 pg/mL	62	60	64	148	Primary accuracy set
Nan 2023 [37]	sFlt-1	<1138.5 pg/mL	54	56	72	152	Not pooled (k < 4 for this marker)
Kim 2017 [34]	PlGF	<0.37 MoM	28	21	72	179	Primary accuracy set
Furuta 2017 [33]	PlGF	10th centile	4	15	9	155	Primary accuracy set
Furuta 2017 [33]	sFlt-1/PlGF ratio	90th centile	6	12	7	158	Not pooled (k < 4 for this marker)
Birdir 2017 [32]	PlGF	34.7 pg/mL	8	8	12	57	Primary accuracy set
Birdir 2017 [32]	sFlt-1/PlGF ratio	78.7 (unitless)	6	4	14	61	Not pooled (k < 4 for this marker)
Lesmes 2015 [30]	PlGF	10th centile	94	923	387	8311	Primary accuracy set
Lesmes 2015 [30]	sFlt-1	10th centile	49	723	327	6516	Not pooled (k < 4 for this marker)

TP, true positive; FP, false positive; FN, false negative; TN, true negative
One operating point per study (Section 4.6). Rows without complete counts (AUC-only reports) were not poolable.

Table S5. Comparison of random-effects methods and 95% prediction intervals for the primary continuous analyses

Marker	k	Random-effects method	τ^2	SMD (95% CI)	P	95% prediction interval
PlGF	19	DerSimonian–Laird	0.126	−0.35 (−0.53 to −0.17)	<0.001	—
PlGF	19	REML (primary)	0.264	−0.38 (−0.63 to −0.13)	0.002	−1.50 to 0.74
PlGF	19	REML + HK	0.264	−0.38 (−0.66 to −0.10)	0.010	—
sFlt-1	12	DerSimonian–Laird	0.044	−0.05 (−0.20 to 0.10)	0.525	—
sFlt-1	12	REML (primary)	0.246	0.03 (−0.27 to 0.34)	0.822	−1.12 to 1.19
sFlt-1	12	REML + HK	0.246	0.03 (−0.35 to 0.42)	0.848	—
sFlt-1/PlGF ratio	7	DerSimonian–Laird	0.059	0.33 (0.11 to 0.55)	0.003	—
sFlt-1/PlGF ratio	7	REML (primary)	0.142	0.39 (0.08 to 0.70)	0.015	−0.66 to 1.44
sFlt-1/PlGF ratio	7	REML + HK	0.142	0.39 (−0.02 to 0.80)	0.060	—

The REML rows correspond to the primary analyses reported in the main text (Figures 2–4). The DerSimonian–Laird and REML with Hartung–Knapp adjustment analyses were performed as sensitivity analyses. The 95% prediction intervals were calculated for the primary REML models using the t distribution with $k - 2$ degrees of freedom. HK, Hartung–Knapp adjustment; REML, restricted maximum likelihood; SMD, standardized mean difference; CI, confidence interval; k, number of studies; τ^2, between-study variance.

Table S6. Restriction sensitivity analyses of the primary continuous syntheses (REML random-effects model)

Marker	Analysis	k	SMD (95% CI)	P	I² (%)
PlGF	Primary analysis (all studies)	19	−0.38 (−0.63 to −0.13)	0.002	92.0
	Excluding Shim 2021 and Rahman 2024	17	−0.41 (−0.69 to −0.13)	0.004	92.8
	Reported mean/SD or direct SE-to-SD conversion	9	−0.37 (−0.52 to −0.22)	<0.001	57.4
	NOS ≥ 7 only	16	−0.42 (−0.59 to −0.25)	<0.001	77.5
	Excluding non-nested case–control designs	14	−0.29 (−0.36 to −0.22)	<0.001	59.1
	Excluding studies with <20 cases	16	−0.23 (−0.42 to −0.04)	0.018	91.9
	By scale: raw concentrations	12	−0.41 (−0.58 to −0.24)	<0.001	75.0
	By scale: multiples of the median	4	−0.31 (−1.48 to 0.86)	0.602	97.9
	By scale: log-transformed	3	−0.34 (−0.42 to −0.26)	<0.001	0.0
sFlt-1	Primary analysis (all studies)	12	0.03 (−0.27 to 0.34)	0.822	79.5
	Excluding Shim 2021 and Rahman 2024	10	0.06 (−0.34 to 0.46)	0.769	82.0
	Reported mean/SD or direct SE-to-SD conversion	7	−0.12 (−0.30 to 0.05)	0.174	58.9
	NOS ≥ 7 only	10	0.08 (−0.29 to 0.46)	0.658	81.3
	Excluding non-nested case–control designs	9	0.11 (−0.33 to 0.55)	0.612	84.5
	Excluding studies with <20 cases	10	−0.14 (−0.23 to −0.04)	0.004	48.0
	By scale: raw concentrations	9	0.18 (−0.22 to 0.59)	0.376	80.1
	By scale: log-transformed	3	−0.29 (−0.50 to −0.07)	0.009	72.6
sFlt-1/PlGF ratio	Primary analysis (all studies)	7	0.39 (0.08 to 0.70)	0.015	80.0
	Excluding Shim 2021 and Rahman 2024	5	0.50 (0.03 to 0.97)	0.038	86.3
	Reported mean/SD or direct SE-to-SD conversion	4	0.42 (0.01 to 0.83)	0.047	84.6
	NOS ≥ 7 only	6	0.47 (0.13 to 0.81)	0.006	79.9
	Excluding non-nested case–control designs	6	0.46 (0.10 to 0.82)	0.011	82.4
	Excluding studies with <20 cases	6	0.29 (0.02 to 0.55)	0.035	76.4

The sampling-window restriction excludes Shim 2021 and Rahman 2024. The mean/SD restriction includes studies reporting means and standard deviations directly or allowing direct SE-to-SD conversion, without median- or IQR-based estimation. "NOS ≥ 7" includes studies rated 7–9 stars. Exclusion of non-nested case–control studies retains nested case–control and cohort designs. The post hoc small-case-group analysis excludes studies with fewer than 20 affected pregnancies. Primary estimates are shown for reference. "By scale" rows present exploratory analyses stratified by reporting scale; the ratio was not stratified because six of seven studies used the unitless ratio scale and only one used an alternative scale. k, number of studies; SMD, standardized mean difference; CI, confidence interval; NOS, Newcastle–Ottawa Scale; SD, standard deviation; SE, standard error.

Table S7. Leave-one-out sensitivity analyses of the primary random-effects (REML) estimates

Marker	Study omitted	k	SMD (95% CI)	P	I² (%)	τ²
PlGF	Ong 2001 [19]	18	−0.41 (−0.56 to −0.26)	<0.001	74.9	0.071
	Tjoa 2001 [20]	18	−0.37 (−0.62 to −0.11)	0.004	92.4	0.272
	Crispi 2008 [23]	18	−0.32 (−0.55 to −0.09)	0.005	91.9	0.208
	Erez 2008 [24]	18	−0.39 (−0.66 to −0.13)	0.003	92.4	0.286
	Cowans 2010 [25]	18	−0.30 (−0.52 to −0.09)	0.005	91.6	0.179
	Steinberg 2010 [26]	18	−0.40 (−0.66 to −0.15)	0.002	92.4	0.277
	Karagiannis 2011 [27]	18	−0.39 (−0.66 to −0.13)	0.004	92.4	0.287
	Ohkuchi 2011 [28]	18	−0.39 (−0.65 to −0.13)	0.004	92.4	0.287
	Ghosh 2013 [29]	18	−0.38 (−0.64 to −0.12)	0.004	92.4	0.284
	Lesmes 2015 [30]	18	−0.39 (−0.65 to −0.12)	0.004	92.2	0.288
	Kienast 2016 [31]	18	−0.37 (−0.62 to −0.11)	0.005	92.2	0.276
	Birdir 2017 [32]	18	−0.38 (−0.64 to −0.12)	0.004	92.4	0.283
	Kim 2017 [34]	18	−0.40 (−0.66 to −0.13)	0.003	92.4	0.285
	Zhang 2019 [35]	18	−0.39 (−0.65 to −0.12)	0.004	92.4	0.288
	Shim 2021 [36]	18	−0.40 (−0.66 to −0.14)	0.003	92.4	0.284
	Nan 2023 [37]	18	−0.38 (−0.65 to −0.12)	0.004	92.3	0.287
	Rahman 2024 [39]	18	−0.40 (−0.66 to −0.13)	0.003	92.3	0.286
	Zhang 2024 [38]	18	−0.37 (−0.62 to −0.11)	0.006	91.7	0.278
	Bai 2025 [40]	18	−0.39 (−0.65 to −0.13)	0.003	92.4	0.286
sFlt-1	Thadhani 2004 [21]	11	0.04 (−0.30 to 0.38)	0.820	81.0	0.298
	Wathen 2006 [22]	11	0.04 (−0.30 to 0.37)	0.823	81.2	0.289
	Crispi 2008 [23]	11	−0.13 (−0.23 to −0.04)	0.004	43.6	0.009
	Erez 2008 [24]	11	0.07 (−0.27 to 0.41)	0.694	81.0	0.293
	Ohkuchi 2011 [28]	11	0.08 (−0.26 to 0.41)	0.649	79.3	0.279
	Lesmes 2015 [30]	11	0.06 (−0.28 to 0.41)	0.731	81.3	0.301
	Kienast 2016 [31]	11	0.04 (−0.30 to 0.38)	0.831	81.0	0.297
	Birdir 2017 [32]	11	0.04 (−0.30 to 0.38)	0.814	81.2	0.295
	Shim 2021 [36]	11	0.04 (−0.31 to 0.38)	0.829	80.7	0.300
	Nan 2023 [37]	11	0.06 (−0.28 to 0.41)	0.710	81.2	0.296
	Rahman 2024 [39]	11	0.06 (−0.29 to 0.40)	0.756	80.9	0.304
	Bai 2025 [40]	11	0.09 (−0.23 to 0.40)	0.589	79.0	0.246
sFlt-1/PlGF ratio	Crispi 2008 [23]	6	0.29 (0.02 to 0.55)	0.035	76.4	0.087
	Ohkuchi 2011 [28]	6	0.47 (0.13 to 0.81)	0.006	79.9	0.139
	Kienast 2016 [31]	6	0.29 (0.01 to 0.56)	0.046	72.3	0.092
	Birdir 2017 [32]	6	0.35 (0.01 to 0.70)	0.045	81.0	0.157
	Shim 2021 [36]	6	0.42 (0.04 to 0.80)	0.030	82.9	0.188
	Nan 2023 [37]	6	0.46 (0.10 to 0.82)	0.011	82.4	0.159
	Rahman 2024 [39]	6	0.45 (0.07 to 0.82)	0.020	83.2	0.179

Each row shows the pooled estimate after omission of the named study, re-estimated using the same estimation and inference methods as in the primary analysis. k, number of remaining studies; SMD, standardized mean difference; CI, confidence interval; I^2, percentage of variability attributable to between-study heterogeneity; τ^2, estimated between-study variance.

Table S8. Sensitivity analyses of the bivariate (Reitsma) diagnostic-accuracy synthesis for PlGF

Analysis	k	Sensitivity (95% CI)	Specificity (95% CI)	LR+	LR−	DOR
Primary (all five studies)	5	0.29 (0.20 to 0.40)	0.86 (0.80 to 0.90)	2.03	0.83	2.45
Excluding Nan 2023 (high QUADAS-2 concerns)	4	0.26 (0.19 to 0.34)	0.90 (0.89 to 0.90)	2.54	0.83	3.07
Excluding Lesmes 2015 (largest study)	4	0.36 (0.26 to 0.46)	0.85 (0.77 to 0.91)	2.45	0.75	3.25

The primary row corresponds to the analysis reported in the main text. Nan 2023 was rated as having high risk-of-bias/applicability concerns in several QUADAS-2 domains (Table S10); Lesmes 2015 is the largest contributing study. LR+, positive likelihood ratio; LR−, negative likelihood ratio; DOR, diagnostic odds ratio.

Table S9. Newcastle–Ottawa Scale assessment of the included studies

Study	Design (NOS version)	Selection (/4)	Comparability (/2)	Outcome/Exposure (/3)	Total (/9)	Notes
Ong 2001 [19]	Case-control (stored serum samples)	3	1	2	6	MoM (GA-controlled); stored-sample representativeness and storage duration not matched
Tjoa 2001 [20]	Cohort (retrospective analysis within a longitudinal study)	3	1	2	6	Small sample (n = 33); limited representativeness
Thadhani 2004 [21]	Nested case-control (MOMS)	4	1	2	7	Nested in prospective cohort; uniform assay
Wathen 2006 [22]	Case-control (stored serum samples)	3	1	2	6	Stored samples; case representativeness unclear
Erez 2008 [24]	Longitudinal case-control	4	2	2	8	Matched for age, BMI, and parity
Crispi 2008 [23]	Nested case-control	4	1	2	7	Small isolated-IUGR subgroup (n = 9)
Cowans 2010 [25]	Matched case-control	4	1	2	7	Very small SGA-without-PE subgroup (n = 8); limited precision
Steinberg 2010 [26]	Nested case-control (MOMS)	4	1	2	7	Nested in prospective cohort
Karagiannis 2011 [27]	Prospective screening cohort	4	2	3	9	Large unselected screening cohort; MoM adjusted for maternal factors
Ohkuchi 2011 [28]	Prospective cohort	3	1	2	6	Some referred patients mixed in; representativeness deducted
Ghosh 2013 [29]	Prospective cohort	4	1	2	7	Unselected antenatal-clinic population
Lesmes 2015 [30]	Prospective screening cohort	4	2	3	9	Large cohort (N = 9,715); MoM adjusted; complete follow-up
Kienast 2016 [31]	Prospective cohort	4	1	2	7	Consecutive routine antenatal enrolment (high-altitude population noted)
Birdir 2017 [32]	Prospective cohort	4	1	2	7	
Furuta 2017 [33]	Prospective cohort	4	1	2	7	GDM/hypertension-excluded healthy cohort (accuracy track only)
Kim 2017 [34]	Prospective cohort	4	1	2	7	
Zhang 2019 [35]	Prospective screening cohort	4	2	2	8	Competing-risks model adjusted for maternal factors
Shim 2021 [36]	Prospective cohort	4	1	2	7	Isolated outcome clear after post hoc PE exclusion
Nan 2023 [37]	Matched case-control	4	1	2	7	Matched design; SGA-only group separated
Rahman 2024 [39]	Population-based cohort	4	2	2	8	Population-based; quartile-adjusted RR; analysis subset of enrollment
Zhang 2024 [38]	Matched case-control	3	2	2	7	1:1 matched on GA and birth date; single-centre retrospective cases
Bai 2025 [40]	Nested case-control	4	1	2	7	Nested in unselected screening cohort

Selection (max 4), Comparability (max 2), Outcome/Exposure (max 3); total max 9. Studies with a score of 7 stars or higher were classified as higher-scoring for descriptive and sensitivity analyses (Section 4.7). NOS totals do not establish uniformly high methodological quality.

Table S10. QUADAS-2 assessment of studies contributing to the diagnostic-accuracy synthesis

Study	D1 RoB	D1 Appl.	D2 RoB	D2 Appl.	D3 RoB	D3 Appl.	D4 RoB	Overall	Notes
Nan 2023 [37]	High	High	Unclear	High	Low	High	Low	High	Matched case-control with composite outcome; SGA-specific 2×2 from supplementary data
Kim 2017 [34]	Low	Low	Low	Low	Low	Low	Low	Low	Prospective cohort; SGA without PE clearly defined
Furuta 2017 [33]	Low	Low	Low	Low	Low	Low	Low	Low	Healthy cohort (GDM/hypertension excluded); centile-based threshold
Birdir 2017 [32]	Low	Low	Unclear	Low	Low	Low	Low	Low	Prospective cohort; PE/PIH explicitly excluded from SGA group
Lesmes 2015 [30]	Low	Low	Low	Low	Low	Low	Low	Low	Large prospective screening cohort (N = 9,715); PE exclusion clear

RoB, risk of bias; Appl., applicability concern. Domains: D1 patient selection, D2 index test, D3 reference standard, D4 flow and timing.

Figure S1. Funnel plots of the primary continuous analyses: PlGF (left), sFlt-1 (center), and the sFlt-1/PlGF ratio (right)

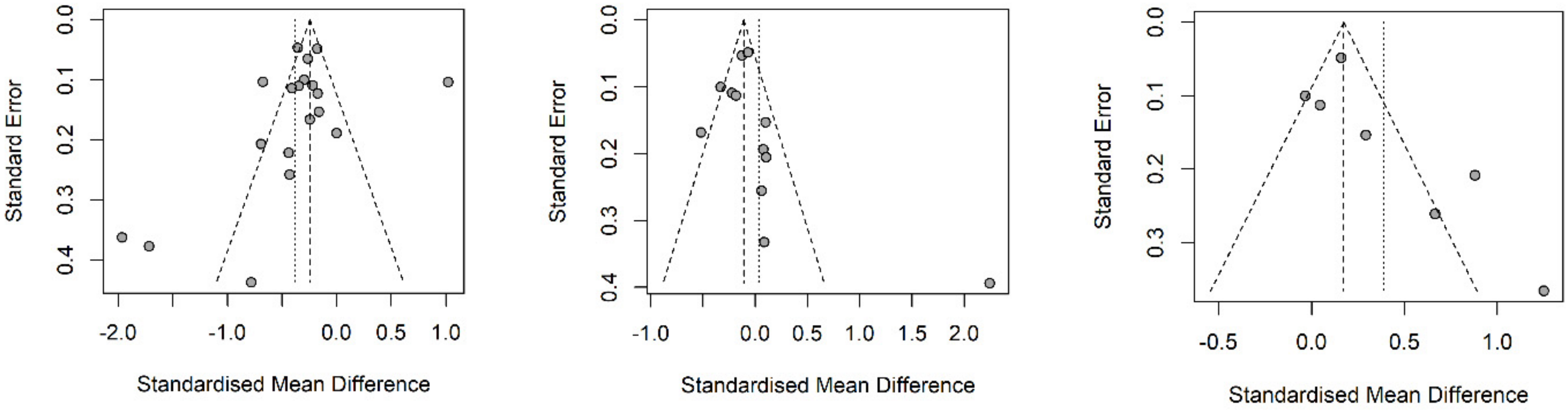

Dashed lines indicate the pseudo 95% confidence region; the vertical dashed and dotted lines mark the random-effects and fixed-effect pooled estimates.
